# PTV2-Fr: a point cloud segmentation network for phenotypic trait extraction and gibberellin effect analysis in sorghum seedlings

**DOI:** 10.3389/fpls.2026.1761249

**Published:** 2026-02-19

**Authors:** Junyi Li, Yunqi Shao, Luxu Tian, Ziyi Zhang, Yurong Guo, Zhibo Zhong, Ruxiao Bai, Peng Yang, Feng Pan, Xiuqing Fu

**Affiliations:** 1 College of Smart Agriculture (College of Artificial Intelligence), Nanjing Agricultural University, Nanjing, China; 2 College of Engineering, Nanjing Agricultural University, Nanjing, China; 3 Institute of Farmland Water Conservancy and Soil-Fertilizer, Xinjiang Academy of Agricultural Reclamation Science, Shihezi, Xinjiang, China; 4 Institute of Mechanical Equipment, Xinjiang Academy of Agricultural Reclamation Science, Shihezi, Xinjiang, China

**Keywords:** gibberellin treatment, phenotypic trait extraction, point cloud segmentation, PTV2-Fr model, sorghum seedlings

## Abstract

Sorghum is a globally important crop. Under the breeding goals of high yield and stress resistance, the precise selection of elite germplasm is crucial. Phenotypic parameters such as plant height and leaf area at the seedling stage are core indicators for evaluating growth vitality. However, traditional manual measurement is inefficient and error-prone, making it difficult to meet the needs of high-throughput research. To address this, this study proposes an improved model (PTV2-Fr) based on Point Transformer V2 (PTV2), which combines 3D point cloud technology to realize the automatic extraction of sorghum seedling phenotypic parameters and explores the regulatory effects of different gibberellin (GA_3_) concentrations. In this study, videos of sorghum seedlings were collected using the relevant system of Nanjing Agricultural University, and reconstructed into.ply format 3D point cloud files via the open-source software Colmap. The core optimizations of the PTV2-Fr model are as follows: Firstly, it proposes a Multi-Radius Dual-Coordinate Attention (MRDCA) mechanism to address the problems of leaf overlap and uneven point cloud density, thereby enhancing feature discrimination ability; Secondly, it introduces a Point-Graph Invariant Feature Refinement (PG-InvFR) module to improve the sensitivity of the segmentation head to local geometric details; Thirdly, it constructs a composite loss function (EL Loss) combining class-weighted cross-entropy loss and Lovász loss to alleviate class imbalance and boost segmentation accuracy. We selected 50 valid datasets from 112 video groups, annotated into three categories: Stem, Leaf, and Pot. The results show that PTV2-Fr outperforms PTV2 by 2.5% in accuracy, with significant improvements in Recall and mean F1-score (mF1). Ablation experiments confirm the positive effects of MRDCA, PG-InvFR, and EL Loss. Furthermore, PTV2-Fr demonstrates good robustness in analyzing GA concentrations, revealing that 50–100 mg/L GA concentrations promote seedling growth, while concentrations exceeding 200 mg/L inhibit growth. The PTV2-Fr model provides an efficient solution for the automatic determination of sorghum seedling phenotypes, and the revealed GA_3_ regulatory mechanism can offer theoretical references for high-quality seedling cultivation and hormone management.

## 1 Introduction

Sorghum is an annual diploid species. As one of the world’s top five food crops, ranking alongside Zea mays, Oryza sativa, Triticum aestivum, and Hordeum vulgare, it serves as a staple food for over 500 million people globally. It is also an important grain and forage dual-purpose crop in arid and semi-arid regions of China and the world, possessing a C4 photosynthetic pathway (Paterson et al., 2009), high water use efficiency (Hossain et al., 2022), and strong drought tolerance (Mwamahonje et al., 2024). It plays a prominent role in ensuring grain and forage supply and developing the bioeconomy in climate-vulnerable regions. To fully exert this core value, breeding elite varieties with strong stress resistance and high productivity is the key path. Phenotypic parameters such as leaf area and stem length are crucial indicators in breeding programs to evaluate the growth vitality of sorghum seedlings and predict variety potential. Traditional phenotypic measurements of seedling traits such as plant height and leaf area often rely on manual determination or low-throughput 2D measurements, which are limited by low efficiency, high labor intensity, significant subjective bias, and insufficient repeatability and traceability (Zhou et al., 2017; Xiang et al., 2021). These limitations make it difficult to meet the needs of large-population verification and high-frequency measurements under multiple treatments. Moreover, the manual measurement process may cause certain damage to plants. For example, measuring stem height with a tape measure may lead to stem bending or even breaking, while leaf area measurement mostly requires detaching the leaves (Gao et al., 2021; Wang et al., 2025). Therefore, there is an urgent need to develop a rapid, accurate, and automated method for the segmentation, extraction, and calculation of sorghum seedling traits.

In the past, computer vision based on 2D images was one of the methods for measuring phenotypic traits of sorghum seedlings (Jingwen and Hong, 2012; Tu et al., 2021; Koyama, 2023). However, as a monocotyledon, sorghum is commonly cultivated with multiple plants per pot, which leads to frequent leaf occlusion between different plants. For 2D computer vision, it is difficult to accurately segment each leaf, resulting in a decrease in segmentation accuracy (Vayssade et al., 2022).

With advances in sensing and computer technologies, high-throughput plant phenotyping (HTPP) has evolved from 2D images to 3D reconstruction and point cloud analysis such as multi-view imaging and lidar (Nguyen et al., 2015; Golbach et al., 2016). Compared with traditional image processing and shallow machine learning, deep learning methods are more promising in terms of robustness to complex backgrounds, end-to-end feature learning, and cross-scene generalization ability, and have become the mainstream approach for seedling phenotypic measurement (Harandi et al., 2023; Mertoğlu et al., 2024; Zhu et al., 2024; Song et al., 2025). In particular, the PointNet algorithm has stood out among a series of vision models due to its ability to directly process sparse and irregular point sets while balancing local and multi-scale geometric features, and serves as a core object detection model in robotics, autonomous driving, and video surveillance (Heiwolt et al., 2021; Boogaard et al., 2022; Li et al., 2022). In the agricultural field, scholars worldwide have specifically optimized the PointNet model to address complex problems in agricultural production. For example, Qiaomei Deng et al (Deng et al., 2024)developed the CPHNet model that can effectively extract stem features of pumpkin seedlings with different shapes, achieving an mean Intersection over Union (mIoU) of 90.4%, mean Precision (mP) of 93.1%, mean Recall (mR) of 95.6%, and mean F1-score (mF1) of 94.4%. Miao, T. et al (Miao et al., 2021)proposed an algorithm for automatically segmenting maize seedling point clouds to separate young maize branches and leaves, with the algorithm’s mP, mR, mF1, and mean Overall Accuracy(mOA) reaching 0.944, 0.956, 0.950, and 0.953, respectively. Jiacheng Shen et al (Shen et al., 2024)lightweighted PointNet++ to segment organ point clouds of cotton seedlings and extract phenotypic traits, with the algorithm’s mP reaching 96.67%. Zhou, Y. et al (Zhou et al., 2025). proposed a non-destructive automatic extraction method for phenotypic traits of Phoebe zhennan seedlings based on 3D point clouds, realizing the extraction of stem and leaf phenotypic parameters through stem-leaf segmentation; the measurement accuracies of stem length, stem diameter, leaf length, leaf width, and leaf area reached 97.7%, 93.2%, 96.4%, 88.02%, and 85.84%, respectively. Liu, Z. et al (Liu et al., 2025)constructed a high-precision organ segmentation network for pumpkin seedling point clouds—FACNet—which achieved 95.06% mIoU, 96.87% mP, 98.02% mR, and 97.44% mF1 on the pumpkin seedling point cloud segmentation dataset. The SN-MGGE network proposed by Yonglong Zhang et al (Zhang et al., 2024). achieved mIoU and OA values of 94.90% and 97.43%, on the cucumber seedling dataset.

Recent advances in plant 3D point cloud analysis have shown a growing interest in deep learning–based segmentation methods tailored for plant phenotyping. A 2025 comprehensive review highlights progress in machine-learning approaches for plant point cloud segmentation, including supervised and unsupervised strategies, and evaluates traditional and deep neural network–based segmentation pipelines such as projection-, voxel-, and point-based models, emphasizing the challenges of data quality, scale variation, and annotation scarcity in plant point clouds (Song et al., 2025). In the context of semantic organ segmentation, recent studies have proposed multi-head hierarchical attention networks and attention-enhanced models that outperform classical feature-based methods, enabling more accurate discrimination of leaves, stems, and other plant organs in diverse environmental conditions (Jin et al., 2025).

In addition to methodological developments, there is active work on improving data resources and task generalization. New annotated datasets provide fine-grained organ-level labels across multiple species, supporting broader benchmarking and evaluation of segmentation models (Gilson et al., 2025). Approaches that aim to retain full resolution without extensive down-sampling, such as species-agnostic sub-sampling strategies, have shown promising results across different sensor modalities and plant types (Galba et al., 2025). Emerging research also explores unsupervised and self-supervised learning to reduce reliance on dense annotations, as well as frameworks for scalable organ segmentation that combine data, algorithmic, and computing perspectives to bridge gaps in practical applications (Du et al., 2025).

In summary, traditional seedling vitality testing cannot meet the needs of agricultural automation due to its shortcomings. Therefore, the future trend is to develop lightweight deep learning network models to rapidly and accurately measure the impacts of different abiotic stresses on seed germination.

Our study proposes a modified model (PTV2-Fr) based on PTV2, which can realize rapid and accurate semantic segmentation of leaves and stems of sorghum seedlings. From the segmentation results, stem length, stem diameter, and leaf area are extracted, providing necessary data support for evaluating the growth status of sorghum seedlings. To summarize, our main contributions are as follows:

1. Dataset construction: A manually annotated sorghum seedling point cloud dataset was established, consisting of 50 pots with 15–25 sorghum seedlings per pot.

2. PTV2-Fr model: A novel point cloud semantic segmentation model (PTV2-Fr) is proposed. Specifically, the model replaces the original grouped vector attention module with MRDCA to enhance the ability to distinguish small organs and cross-scale structures of seedlings; introduces the PG-InvFR module between the decoder output and the segmentation head, which dynamically adapts weights for different spatial positions to further refine local point cloud features; and adopts the composite loss function EL Loss at the loss function level to alleviate class imbalance and directly improve the segmentation accuracy of IoU, boundaries, and small objects.

3. Provides a method for measuring stem length, stem diameter, and leaf area using point cloud data.

4. Sorghum seedling growth experiment: We conducted sorghum seedling growth experiments under different GA_3_ concentration conditions to analyze the effects of hormone treatment on germination potential and early growth patterns. Numerous existing studies have indicated that GA_3_ within an appropriate range can promote germination and seedling elongation, but excessive concentrations or specific stress scenarios may lead to a dose-response phenomenon of “high-concentration promotion and low-concentration inhibition”. For sorghum and related materials such as sweet sorghum, the common effective concentration range is tens to hundreds of mg·L^-^¹ or around 100 μM, with the optimal value varying by genotype and environment. Our experiment set up concentration gradients within this empirical range to characterize the temporal evolution trajectories of key seedling traits under different treatments.

## 2 Materials and methods

### 2.1 Experimental equipment and experimental design

**Figure 1:**
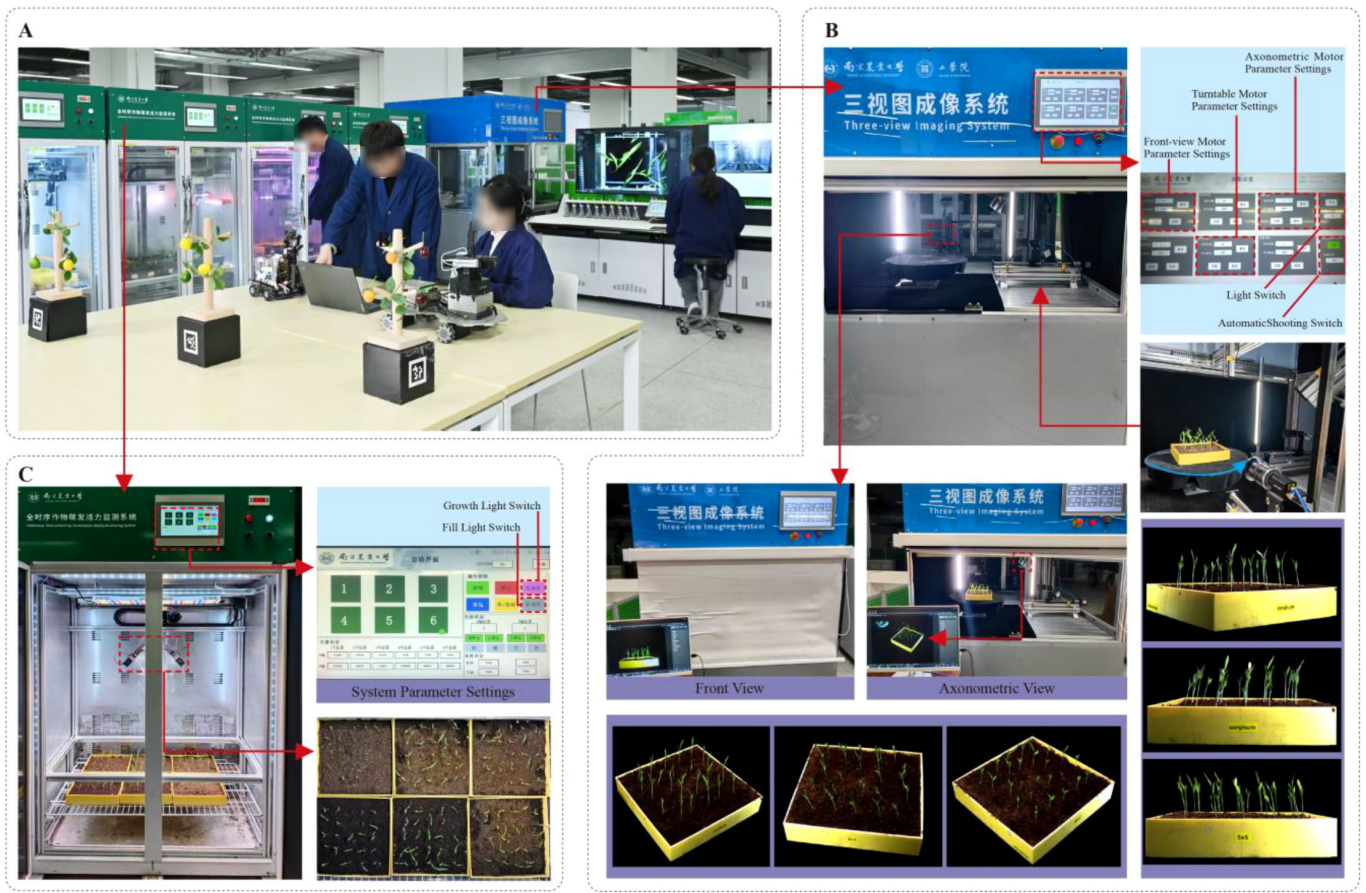
**(A)** Experimental scenario; **(B)** Cultivating sorghum seedlings; **(C)** Three-view imaging.

The Crop Growth and Cultivation System constructed by us consists of a cultivation room environment control module and a rail-based image acquisition module. The overall structure of the Three-View Imaging System is built with aluminum profiles, and the specific configurations are shown in **Tables 1**, **2**. The experimental system process from crop cultivation to image acquisition and growth monitoring is shown in **Figure 1**.

**Table 1:** Crop growth and cultivation system configurations.

Module name	Component name	Functional purpose
Cultivation Room Environmental Control Module	Cultivation Box	Overall bearing of cultivation, environmental control, and image - acquisition related components. Provides a closed cultivation space for crop growth.
	Touch Screen	Installed on the top of the cultivation box for system operation and parameter setting.
	Control Buttons	Integrated on the upper right of the cultivation box, responsible for power control, LED light source switching, and manual temperature adjustment.
	Perforated Partition	Installed inside the cultivation box to divide the box into upper and lower layers for space - partitioned utilization.
	Acrylic Culture Tray	A total of 6, placed in the upper layer of the cultivation box to carry crops for cultivation.
	Deionized Water Storage Area	Located in the lower layer of the cultivation box to store deionized water required for experiments.
	Embedded PTC Hot Air Circulation System	Installed on the side of the cultivation box. Operates to increase the temperature when the temperature is lower than the preset value and stops when it exceeds the upper limit to maintain temperature stability.
	Tp -100 Thermocouple	Installed on the side of the cultivation box to monitor the chamber temperature in real - time.
	LED Light Source	Installed on the side of the cultivation box to supplement lighting for crop growth.
Orbital Image Acquisition Module	HIV VISION RGB Industrial Camera	Used for image acquisition of crop growth status.
	Telecentric Lens	Used in conjunction with the industrial camera to optimize the image acquisition effect.

**Table 2:** Three-view imaging system configurations.

Module name	Component name	Functional purpose
Overall Frame and Support Structure	Aluminum Profile Frame	Builds the overall support structure of the system, providing a stable installation foundation and an effective acquisition space.
Imaging and Sensing Device	Color Camera(MV-CS200-10GC)	Core imaging component for capturing high-resolution RGB images of sorghum seedlings (5472 × 3648 pixels) for multi-view 3D reconstruction.
Motion - Drive Component	Ball Screw Module	Adjusts the spatial position of the camera to meet multi - angle imaging requirements.
Acquisition Platform	Electric Laser Rotary Table	Carries sorghum seedlings and rotates in conjunction with the camera to achieve multi - angle non - destructive image acquisition.
Control Device	Customized Control Panel	Centralized control of the camera and rotary table operation to ensure accurate multi - angle (front view, side view, top view, axonometric view) acquisition.

**Figure 2:**
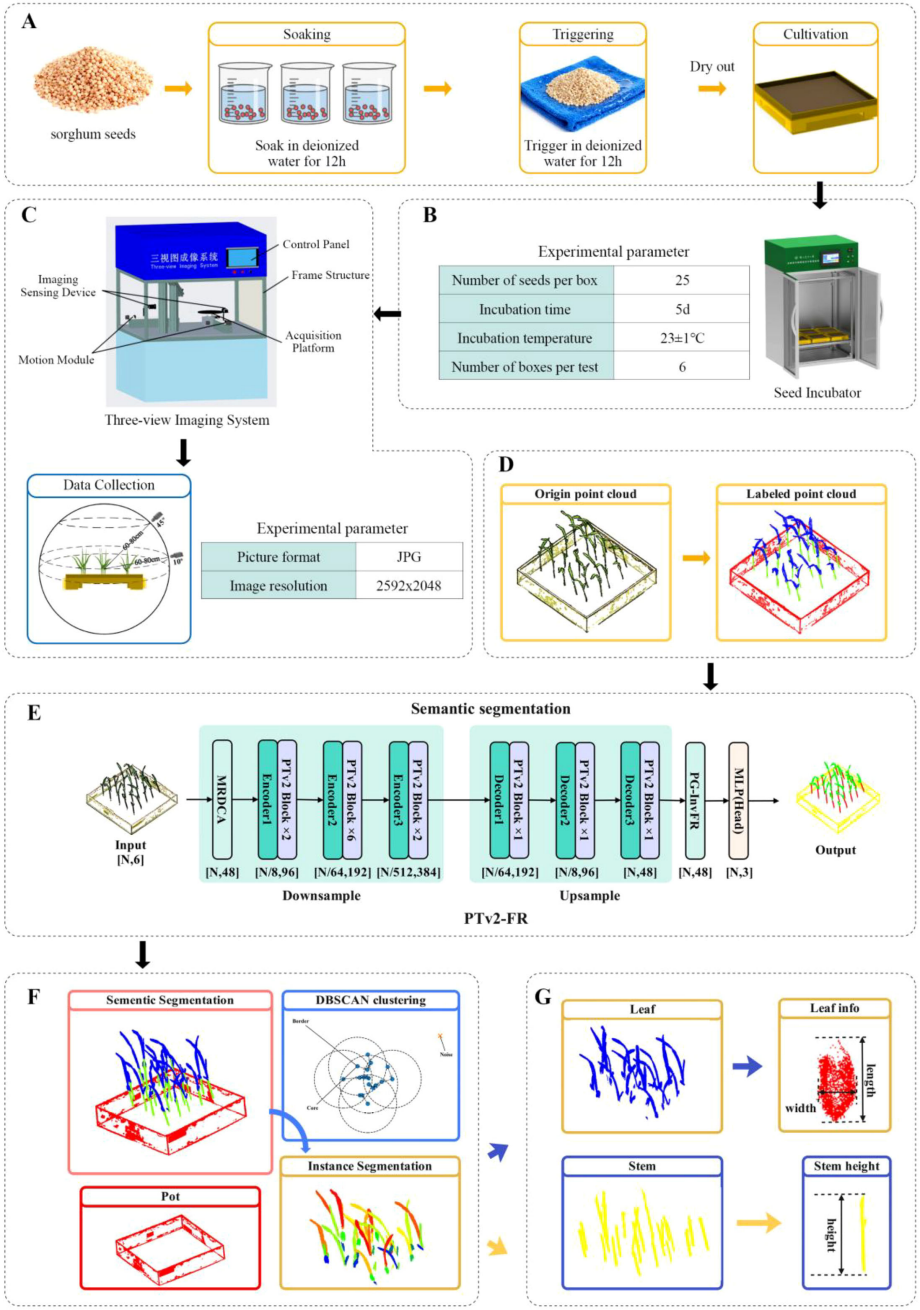
**(A)** Seed pretreatment; **(B)** Crop growth cultivation system; **(C)** Three-view imaging system; **(D)** Data annotation; **(E)** The PTV2-Fr overview; **(F)** Instance segmentation; **(G)** Phenotypic trait extraction.

The structural framework proposed in this paper for acquiring phenotypic information of sorghum seedlings consists of six distinct components, as shown in **Figure 2**. Firstly, Part (A) is the growth and cultivation of sorghum seedlings. Secondly, in Part (B), the cultivation of sorghum seedlings primarily involves setting appropriate parameters using the crop growth cultivation system for cultivation. Thirdly, in Part (C), the acquisition of sorghum point cloud data mainly involves video shooting and 3D reconstruction using the three-view imaging system. Fourthly, in Part (D), the point clouds corresponding to stems, leaves, and pots are annotated. Fifthly, in Part (E), PTV2-Fr is used for semantic segmentation of stems, leaves, and pots. Sixthly, in Part (F), the DBSCAN algorithm is utilized to perform instance segmentation on the sorghum point cloud data after semantic segmentation. Finally, in Part (G), four key phenotypic traits are extracted from the results: stem length, stem diameter, leaf length and leaf width.

### 2.2 Seedling phenotypic image acquisition and dataset construction

We focused on the sorghum seedling stage, sowing 25 Nuoyou No. 1 sorghum seeds in a 5×5 grid in each acrylic culture tray (25 cm in length, 25 cm in width, and 5 cm in height). To ensure the efficiency of subsequent training, the data were collected from a total of 112 video groups of sorghum seedlings before the germination of the third leaf. After manual screening, 50 valid video groups were selected for further processing. These video groups were reconstructed into 3D point clouds using Colmap, based on two perspectives (front view and axonometric view). We generated.ply files in Colmap using two perspectives (axonometric view and front view), and manually annotated these 50 files with CloudCompare to produce 50 high-quality point cloud datasets. Point-level semantic annotation was performed manually using CloudCompare. Annotators labeled points into three classes (leaf, stem, and pot) by interactively selecting regions in 3D space from multiple viewpoints. To improve annotation consistency, a unified annotation guideline was established prior to labeling, particularly for stem–leaf junctions and densely occluded regions. For ambiguous boundary regions where leaves partially overlapped with stems, labels were assigned based on the dominant anatomical structure in the local neighborhood rather than isolated points. All annotated samples were visually inspected after labeling, and inconsistent annotations were corrected through cross-checking by a second annotator. Ambiguous boundary points were labeled conservatively to minimize noise propagation in model training. To enrich data diversity and improve training accuracy, sorghum seedling leaves were annotated as “leaf”, stems as “stem”, and acrylic culture trays as “pot”. To reduce computational complexity, all point clouds of acrylic culture trays were downsampled to 4096 points using Furthest Point Sampling (FPS), which helped retain key features while simplifying the data. This sampling resolution was chosen as a trade-off between preserving fine-grained geometric details of seedling organs and maintaining computational efficiency for transformer-based networks. Preliminary experiments showed that using fewer points led to noticeable degradation in stem–leaf boundary segmentation, whereas higher point counts provided marginal performance gains at a substantially increased computational cost. Importantly, the pot (i.e., the physical cultivation unit) is defined as the fundamental unit for dataset partitioning. Although each pot was scanned at multiple time points, all point clouds originating from the same pot (i.e., all time points and derived point blocks) were assigned to the same data subset to avoid temporal data leakage. The dataset was therefore partitioned at the pot level into training, validation, and test sets with an approximate ratio of 7:2:1 (pots), respectively. This pot-level grouping ensures that the model is evaluated on entirely unseen physical plants.

### 2.3 PTV2-Fr sorghum seedling semantic segmentation design

**Figure 3:**
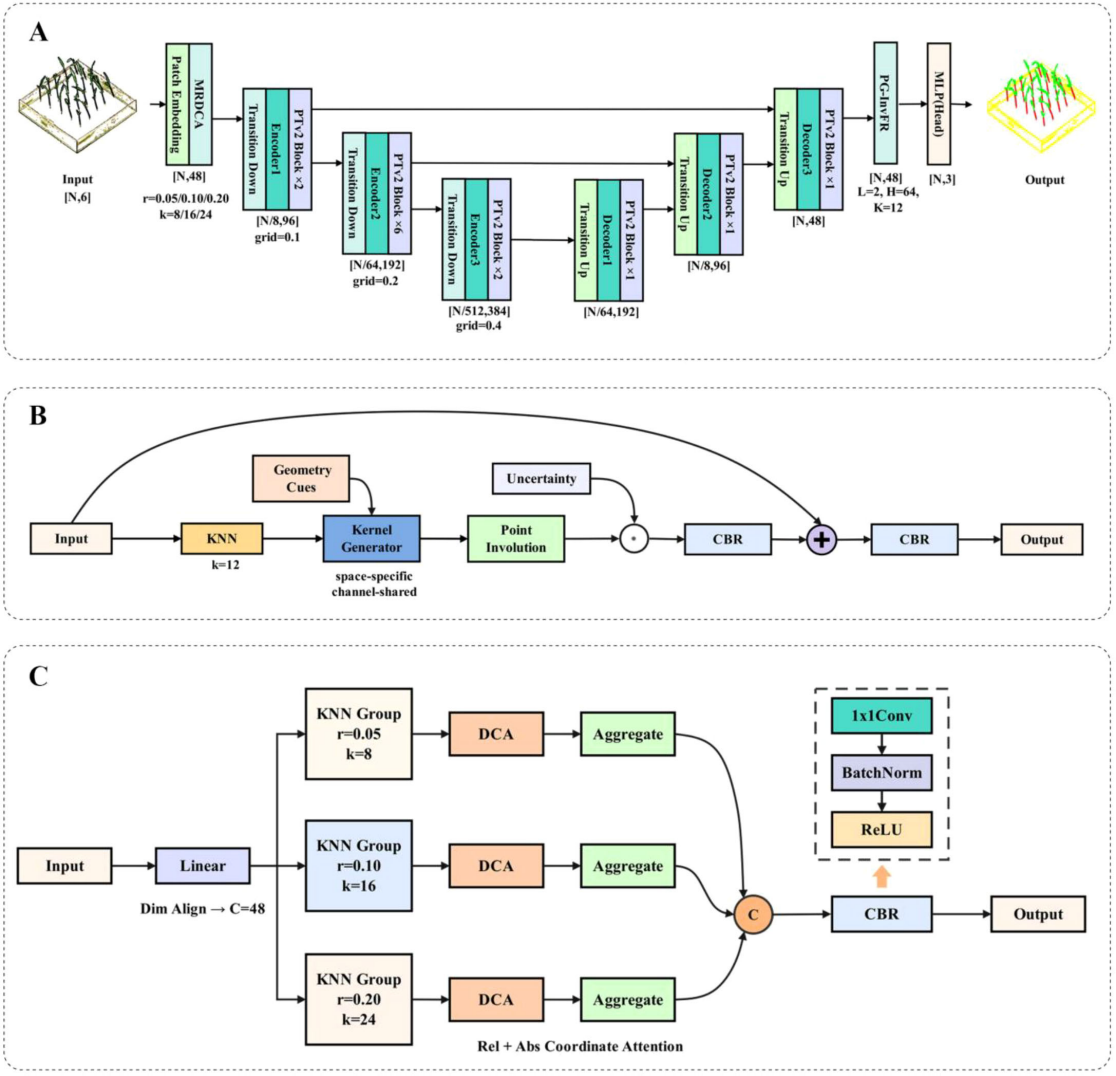
**(A)** The PTV2-Fr architecture; **(B)** The PG-InvFR architecture; **(C)** The MRDCA architecture.

This paper introduces PTV2-Fr, a point cloud semantic segmentation network built on the enhanced PTV2 backbone, specifically designed for the task of distinguishing plant organs in sorghum seedling point clouds. The overall architecture of PTV2-Fr is shown in (**Figure 3A**).

In the feature extraction stage, the MRDCA module is proposed to enhance local geometry and coordinate awareness. This module first learns fine-grained, medium-scale, and coarse-scale patch feature representations through parallel multi-radius branches. Then, the outputs of each branch are fused by concatenation or summation and input into the improved Dual-Coordinate Attention mechanism, which integrates relative positions, normalized absolute coordinates, and channel statistics (avg/max pooling). Adaptive weighting is achieved through learnable gating, significantly improving the model’s ability to distinguish subtle structures, such as leaf edges and leaf-stem junctions.

In the decoding output stage, the model incorporates the PG-InvFR module, which iteratively refines neighborhood features before the segmentation head, effectively strengthening the boundary representation between leaves and stems. To improve the model’s robustness, the training process includes point cloud data augmentation, to address the class imbalance issue and directly optimize the IoU metric, thereby improving the overall robustness and segmentation accuracy of the model.

#### 2.3.1 Point-graph involution feature refinement

In plant point cloud segmentation tasks, PTV2 lacks a feature refinement mechanism for “category boundary gradient” regions, leading to misclassification in fuzzy areas such as the leaf-stem junction, leaf edges, and flower pot edges. To address this issue, the Flora-NET model proposed by Gupta et al. is adopted (Gupta and Tripathi, 2025). This model introduces involution into flower image classification and designs the Involution-Based Feature Refinement(Inv-FR) module. The model performs hierarchical feature refinement on image grids using “spatially specific, channel-independent” dynamic kernels, which enhances the expression of petal boundaries and fine-grained structures in complex backgrounds.

However, there are significant differences between point cloud data and images: point clouds have uneven density, and organ boundaries often exhibit continuous gradients, while the geometric anisotropy and semantic uncertainty of organs such as leaves, petioles, and stems are more pronounced. Directly transferring the Inv-FR module from image-based scenarios cannot fully adapt to point clouds. Therefore, an improved version of InvFR for point clouds, called PG-InvFR, is proposed. This version introduces geometric directional constraints, allowing the dynamic kernel to capture directional features in the point cloud neighborhood, especially in areas like leaf edges and organ transition zones. Additionally, the module incorporates boundary uncertainty modulation, enabling adaptive adjustment of kernel weights in ambiguous regions, which enhances robustness at category transition points. Furthermore, the original grid-based neighborhood is replaced with a k-NN-based point cloud neighborhood graph, ensuring stable feature refinement even with sparse and irregular sampling (Qi et al., 2017a).

Furthermore, beyond replacing the original grid-based neighborhood with a k-NN point cloud graph, PG-InvFR introduces point-cloud-specific refinements: the dynamic kernel generation function is additionally conditioned on directional geometric cues (e.g., distances and local orientation), and an uncertainty-aware modulation term scales the refinement strength in ambiguous boundary regions. These extensions make PG-InvFR a point-cloud-oriented refinement module rather than a simple graph-based adaptation of the original Inv-FR. Through these improvements, the PG-InvFR module not only inherits the efficient dynamic modeling advantages of involution in Flora-NET but also achieves more robust boundary segmentation performance tailored for plant point clouds. It supports sorghum seedling stem-leaf segmentation and parameter extraction. The architecture of PG-InvFR is shown in (**Figure 3B**).

Involution, unlike traditional convolutions, utilizes spatially varying kernels that are independent of the channel dimension. Specifically, the kernel $H_{i}$ is determined by the following dynamic function, as shown in Equation 1:

(1) $$\begin{array}{c} H_{i}=\varphi \left(F_{i}\right)\in R^{K\times K} \end{array}$$

Here, $\;F_{i}$ denotes the local feature at position   i , and φ(·) is the dynamic kernel generation function.

The final output feature is obtained as shown in Equation 2:

(2) $$\begin{array}{c} F_{i}^{\text{out}}=\sum_{\left(m,n\right)\in \text{Δ}K}H_{i}\left(m,n\right)\cdot F_{i+\left(m,n\right)} \end{array}$$

Where  ΔK  is a 2D grid region, indicating the positions of the different kernels applied at various locations.

For the point cloud, we replace the traditional grid-based neighborhood with a k-NN (k-Nearest Neighbors) neighborhood, as shown in Equation 3:

(3) $$\begin{array}{c} N\left(i\right)=\{j|\left|p_{i}-p_{j}\right|<r\} \end{array}$$

Where $p_{i}$ and $p_{j}$ are the coordinates of the points i and j, respectively, and  r defines the neighborhood radius.

In the point cloud neighborhood graph, normal vectors and principal curvature directions are introduced to modulate the kernel weight generation function, as shown in Equation 4:

(4) $$\begin{array}{c} H_{i}=\varphi \left(F_{i},d_{i}\right)=M_{k}\left(M_{c}\left(\left[F_{i},d_{i}\right]\right)\right) \end{array}$$

Where $d_{i}$ is the distance information from point i, and $M_{k}$ and $M_{c}$ are transformation functions applied to the feature and distance information.

This enables the dynamic kernel to adapt to geometric differences in different directions.

Based on the class probability distribution entropy of the neighboring points, the uncertainty measure $U_{i}$ for each point is defined as shown in Equation 5:

(5) $$\begin{array}{c} U_{i}=-\sum_{c}p_{i,c}\log p_{i,c} \end{array}$$

Where $p_{i,c}$ represents the probability of the  i-th point belonging to class  c.

To enhance the adaptability of the kernel in uncertain regions, we introduce the uncertainty weight $\left({\text{α}}_{i}\right)$, which is derived by applying the sigmoid function to the entropy, as shown in Equation 6:

(6) $$\begin{array}{c} {\text{α}}_{i}=\sigma \left(U_{i}\right) \end{array}$$

Where σ(·) is the sigmoid function.

The final output feature $F_{i}^{\text{out}}$ is computed with a weighted sum, where the weight is determined by the uncertainty weight $\alpha_{i}$ and the relationship matrix $H_{i,j}$ between neighboring points, as shown in Equation 7:

(7) $$\begin{array}{c} F_{i}^{\text{out}}={\text{α}}_{i}\sum_{j\in N\left(i\right)}H_{i,j}\cdot f_{j} \end{array}$$

Here, $f_{j}$ is the feature of the   j-th point, and N(i) denotes the neighborhood of the  i-th point.

Here, the original grid ΔK  is replaced by a k-NN/ball neighborhood-based point cloud graph to ensure stable kernel behavior under sparse and irregular sampling conditions. The final output feature is as shown in Equation 8:

(8) $$\begin{array}{c} \;\;f_{i}^{\text{out}}={\text{α}}_{i}\sum_{j\in N\left(i\right)}H_{i,j}\cdot f_{j} \end{array}$$

Thus, when the uncertainty is high, the network increases the adaptability of the dynamic kernel, thereby reducing boundary misclassification.

#### 2.3.2 Multi-radius dual-coordinate attention

In sorghum seedling point clo organ segmentation, challenges such as overlapping leaves, uneven point density, and significant organ scale differences arise. While the original attention mechanism of PTV2 performs excellently in general point cloud tasks, it faces structural limitations when directly applied to seedling point clouds (Chu et al., 2024; Zhou et al., 2024). Specifically, in scenes with uneven point density and prominent organ scale differences, this mechanism can confuse features from different leaves or the leaf-stem junction. Short-range neighborhoods struggle to capture global semantics across leaves, while long-range neighborhoods may overwhelm fine boundary information, resulting in blurred boundaries and misclassification. Furthermore, although the Relative Positional Encoding (RPE) in PTV2 provides positional information for attention, its single encoding form is often insufficient to express complex geometric differences in highly localized geometric changes, such as overlapping leaves and fine stems. This leads to an inability to fully highlight subtle but discriminative relative positional features.

To address these issues, we propose the MRDCA module to enhance local geometry and coordinate awareness. The architecture of MRDCA is shown in (**Figure 3C**). Compared with existing multi-scale or coordinate attention mechanisms used in point-based transformers, MRDCA introduces several design differences tailored to 3D plant point clouds. Most prior multi-scale attention modules rely on fixed receptive fields and stack multiple independent blocks, or simply concatenate features computed with different neighborhood sizes, while coordinate information is injected through a single positional encoding stream. In contrast, MRDCA realizes multi-radius modeling inside one unified module by constructing parallel local branches with different radii/neighbor sizes and dynamically aggregating them through learnable gates. Moreover, instead of mixing geometric cues in one branch, MRDCA explicitly separates relative geometric offsets, absolute 3D coordinates, and channel-wise feature statistics into distinct projections before fusion. This decoupled design allows the attention mechanism to emphasize subtle but discriminative positional cues such as leaf tips and leaf–stem junctions, and to better handle highly non-uniform point densities that are characteristic of crop canopies.

In the patch embedding stage, parallel branches with different receptive fields (defined by different radii or the number of neighbors K) are constructed to capture geometric features at various scales. These features include local features for leaf edges and fine stems, as well as global features for inter-leaf relationships and context. The multi-scale information is then integrated into the subsequent Transformer module using strategies like concatenation or summation. The input point features are first dimensionally aligned as shown in Equation 9:

(9) $$\begin{array}{c} f^{\text{proj}}=\text{ReLU}\left(\text{PointBN}\left({\text{Linear}}_{\text{proj}}\left(f\right)\right)\right) \end{array}$$

After alignment, $f^{\text{proj}}$ is divided into three branches (fine/medium/coarse, each with 16 channels), corresponding to short/medium/long receptive fields. Each branch constructs a KNN neighborhood with a different neighborhood size ( e.g., ( K1 = 8, K2 = 16, K3 = 24 )) and performs feature embedding and aggregation within its respective neighborhood as shown in Equation 10:

(10) $$\begin{array}{c} F_{\text{mr}}=\underset{r\in \left\{r_{s},r_{m},r_{l}\right\}}{⊕}{\text{Embed}}_{r}\left(f^{\text{proj}}\right) \end{array}$$

Where ⊕ represents concatenation or summation across the channels.

Dual-Coordinate Attention (DCA) computes neighborhood attention by simultaneously utilizing fine-grained relative features (rel), normalized absolute coordinate encoding (abs), and channel-level statistics (avg/max pooling). It adaptively assigns weights between these signals via learnable gates (pool_gate/gate). This enables the model to amplify small but discriminative relative position differences (e.g., subtle displacements at the leaf-stem junction) while using absolute positional information within instances to avoid mis-clustering leaves due to local similarities.

For point  i  in the branch and its neighbor   j , we define as shown in Equations 11 and 12:

(11) $$\begin{array}{c} q_{i}=\varphi \left(W_{q}f_{i}\right),\;k_{j}=\varphi \left(W_{k}f_{j}\right),\;v_{j}=W_{v}f_{j} \end{array}$$

(12) $$\begin{array}{c} \Delta p_{ij}=p_{j}-p_{i},\;\Delta f_{ij}=f_{j}-f_{i},\;p_{i}^{\text{norm}}=\frac{p_{i}-\min\left(p\right)}{\max\left(p\right)-\min\left(p\right)} \end{array}$$

The attention weights are computed as shown in Equations 13 and 14:

(13) $$\begin{array}{c} w_{ij}={\text{Softmax}}_{j}\left(\frac{q_{i}k_{j}^{T}}{\sqrt{d}}+{\text{ψ}}_{\text{rel}}\left(\text{Δ}p_{ij},\text{Δ}f_{ij}\right)+{\text{ψ}}_{\text{abs}}\left(p_{i}^{\text{norm}}\right)+{\text{αψ}}_{\text{pool}}\left(\text{Δ}f_{ij}\right)\right) \end{array}$$

The weighted sum of features is then computed as:

(14) $$\begin{array}{c} \;\;f_{i}^{\text{enh}}=\sum_{j\in N_{k}\left(i\right)}w_{ij}v_{j} \end{array}$$

Where ${\text{ψ}}_{\text{rel}}$, ${\text{ψ}}_{\text{abs}}$, and ${\text{ψ}}_{\text{pool}}$ represent MLP projections for relative position, absolute coordinates, and pooling operations, respectively.  α is a learnable parameter, and φ=ReLU∘PointBN.

The enhanced features from the three branches are concatenated to form a multi-scale representation and optionally aligned by a linear layer before being fed into the backbone/decoder, as shown in Equations 15 and 16:

(15) $$\begin{array}{c} f^{\text{MRDCA}}=\text{Concat}\left(f_{\left(r_{s}\right)}^{\text{enh}},f_{\left(r_{m}\right)}^{\text{enh}},f_{\left(r_{l}\right)}^{\text{enh}}\right) \end{array}$$

(16) $$\begin{array}{c} \;\;f^{\text{pre}}=\varphi \left(W_{\text{fuse}}f^{\text{MRDCA}}\right) \end{array}$$

#### 2.3.3 EL loss

To address the challenges of class imbalance (e.g., low proportion of stem samples), blurry boundary prediction, and small target misdetection in plant point cloud segmentation tasks, this paper designs a hybrid loss function, EL Loss, which combines Weighted Cross-Entropy Loss and Multiclass Lovász Loss (Berman et al., 2018). By jointly optimizing class balance and boundary precision, EL Loss improves the model’s segmentation performance for fine categories, such as plant stems and flower pots.

The Weighted Cross-Entropy Loss is based on the standard cross-entropy loss, with a learnable weight coefficient introduced for each class to alleviate the model bias caused by class imbalance in the dataset. To mitigate class imbalance, class weights were computed based on the point distribution after FPS downsampling. Specifically, the proportion of each semantic class was calculated using the downsampled training point clouds, and inverse-frequency weighting was applied during loss computation.

Compared to cross-entropy loss, Lovász Loss focuses on the probability error of individual points. By minimizing the Lovász distance between the “prediction error set” and the “true class set,” it emphasizes the overall consistency of the segmentation regions. This is especially helpful for improving the blurry boundary issue (e.g., between the leaf and stem junction or the edges of flower pots), while also increasing the recall of small targets (e.g., fine stems).

To leverage both the “class balancing ability” of the Weighted Cross-Entropy Loss and the “boundary optimization ability” of Lovász Loss, this paper linearly combines the two losses with a specific weight ratio, forming the final hybrid loss function, as shown in Equation 17.

(17) $$\begin{array}{c} L_{\text{EL}}=\alpha L_{\text{CE}}+\beta L_{\text{Lovász}} \end{array}$$

Where $L_{\text{CE}}$ is the Weighted Cross-Entropy Loss, $L_{\text{Lovász}}$ is the Lovász Loss, and α and β are hyperparameters that control the relative weights of the two loss functions.

#### 2.3.4 Phenotypic trait extraction

The raw point cloud is obtained from the 3D scanning of the plant. Due to uniform scaling biases in the reconstruction coordinates, the three axes are first scaled by a constant factor. All subsequent distance thresholds are set based on the real scale (in meters) after scaling. Using color coding for bitwise matching, three subsets are obtained: stem points, leaf points (the base points are excluded from the analysis).

Project the point cloud onto the xy plane and use DBSCAN to perform density-based clustering. Let ϵ be the radius, and minPts be the minimum number of neighboring points. The ϵ-neighborhood of point $p_{i}$ is defined as shown in Equation 18:

(18) $$\begin{array}{c} N_{\epsilon }\left(p_{i}\right)=\{p_{j}||p_{j}-p_{i}{|}_{2}\leq \epsilon \} \end{array}$$

When $\left|N_{\epsilon }\left(p_{i}\right)\right|\geq \text{minPts}$, $p_{i}$ is considered a core point, and density-reachable relationships form a cluster. Each cluster is treated as a “cluster point”, and points labeled as -1 are deleted. To match the experimental scale, the coordinates are scaled to actual sizes, and the default interval is set to $s_{\text{stem}}=0.03\text{ m}$ (3 cm), with minPts=80. For each plant c, the distance to the centroid, ${\text{μ}}_{\text{stem}}\in R^{2}$, is recorded as a parameter associated with the cluster point.

In the RANSAC Line Fitting process, two points, $p_{0}$ and $p_{1}$, are randomly selected to define the direction v of the line. The distance from each point to the line is calculated using the formula, as shown in Equation 19:

(19) $$\begin{array}{c} d\left(x\right)=\left||\left(x-p_{0}\right)\times \hat{v}|\right|,\;\hat{v}=\frac{v}{\left||v|\right|} \end{array}$$

Here, d(x) represents the perpendicular distance from a point xxx to the line defined by $p_{0}$ and $p_{1}$. The point distance threshold is set to τ=0.01 cm, and the algorithm is run for 120 iterations, after which the most frequent model is selected as the final fit.

In the PCA Precision step, Singular Value Decomposition (SVD) is performed on the set of points, centered around their mean position using the function. The first principal component $a_{c}$ is chosen as the direction of maximum variance, with the constraint that $a_{c,z}\geq 0$. The points are then centered around ${\bar{p}}_{c}$, ensuring that the data points are aligned to maximize the direction of variance.

To avoid cross-plant connections, all leaf points are first assigned to the corresponding plant c based on the closest stem cluster centroid. This ensures that the leaf processing within each plant does not interfere with others. For each plant c‘s set of leaf points, PCA and Delaunay triangulation are used to estimate the geometric properties:

For leaf length/width, PCA is applied to the points, where the first and third principal components $u_{1},u_{2}$ are used to project the points onto the principal axes. The leaf’s length and width are then computed as the differences between the maximum and minimum projections along the $u_{1}$ and $u_{2}$ axes, as shown in Equation 20:

(20) $$\begin{array}{c} L=\max\left\{{\text{π}}_{1}\right\}-\min\left\{{\text{π}}_{1},\right\}\;\circ W=\max\left\{{\text{π}}_{2}\right\}-\min\left\{{\text{π}}_{2}\right\} \end{array}$$

For leaf area, the Delaunay triangulation is performed on the points projected onto the $\left(\pi_{1},\pi_{2}\right)$ plane. The area of each triangle in the triangulation is calculated using the formula for the surface area of a triangle in 3D space, as shown in Equation 21:

(21) $$\begin{array}{c} A=\sum_{\text{Δ}\left(i,j,k\right)}\frac{1}{2}\left||\left(p_{j}-p_{i}\right)\times \left(p_{k}-p_{i}\right)|\right| \end{array}$$

Here, the summation is over the triangles formed by the points, and $p_{i},p_{j},p_{k}$ are the vertices of the triangle. This calculation gives the total area of the leaf projected onto the plane.

This approach is robust to slight wrinkling and yields a 3D leaf area closer to the actual measurement than a direct 2D area estimate.

#### 2.3.5 Model evaluation metrics

To assess the effectiveness of semantic segmentation, the following metrics are used: Intersection over Union (IoU), Precision, Recall, and F1 score. These metrics provide a multi-dimensional evaluation of the model’s performance, ensuring the reliability and comprehensiveness of the evaluation, as shown in Equations 22–25.

(22) $$\begin{array}{c} Precision=\frac{TP}{TP+FP} \end{array}$$

(23) $$\begin{array}{c} Recall=\frac{TP}{TP+FN} \end{array}$$

(24) $$\begin{array}{c} F1-score=2\times \frac{Precision\times Recall}{Precision+Recall} \end{array}$$

(25) $$\begin{array}{c} IoU=\frac{TP}{TP+FP+FN} \end{array}$$

Where TP, FP, and FN represent true positive, false positive, and false negative, respectively.

## 3 Results

### 3.1 Operating environment

To ensure that the results of PTV2-Fr are not affected by different experimental conditions, all experiments in this study were conducted on an Ubuntu 18.04 server equipped with 120 vCPUs (AMD EPYC 7642 48-Core Processor). Acceleration was achieved using an NVIDIA RTX 3090 GPU with 24GB of video memory. The PTV2-Fr method was implemented based on the CUDA 11.3 + PyTorch 1.12.1 framework. **Table 3** presents the various system configurations used for experimental simulation.

**Table 3:** Configuration of the experimental simulation system.

Hardware configuration	Software configuration
RAM:32GB	OS: Ubuntu 18.04
CPU: 120 vCPU (AMD EPYC 7642 48-Core Processor)	Pytorch: 1.12.1
GPU: NVIDIA RTX 3090 (24GB)	CUDA version:11.3
Memory: 90GB	Python: 3.8.20

For implementation with the Pointcept framework, the dataset files are organized using an S3DISDataset-style directory convention. We emphasize that the “Area_1–Area_6” labels are an implementation-level organization scheme and do not represent literal spatial regions in the experiment. Each Area corresponds to a fixed subset of pots (i.e., complete pot-level groups including all time points). During all experiments, the mapping from pots to Areas was fixed, and all time points from the same pot were mapped to the same Area. The target classes are three semantic labels, with the ignored label set to -1. The data root directory is data/train_2, with the target categories clearly defined as three classes: leaf, stem, and pot, and the ignored label set to -1. During the model training phase, the dataset was divided into training, validation, and test sets at a ratio of 7:2:1. The training data included 50 annotated point cloud files categorized into three classes (leaf, stem, and pot), corresponding to 35 pots in the training set, 10 pots in the validation set, and 5 pots in the test set. The training data consisted of 50 point cloud files, annotated into three categories: leaf, stem, and pot. We used a batch size of 32 and trained the model for 100 epochs. The AdamW optimizer was applied with a learning rate of 0.001 and weight decay of 0.05. The learning rate was adjusted using a MultiStepLR scheduler, decaying by a factor of 0.05 after 60 and 80 epochs. To enhance the generalization ability and robustness of the model, a number of targeted data augmentation techniques have been integrated during the training process, as detailed in **Table 4**.

**Table 4:** Data augmentation techniques used in training.

Augmentation method	Specific parameters	Core function
RandomScale	Randomly scale the scale to 0.9-1.1 times	Simulate target scale changes caused by different distances during point cloud acquisition and improve the model’s adaptability to scale differences.
RandomFlip	Perform random flipping with a probability of 0.5	Enhance the model’s ability to recognize targets in different postures through symmetric transformation.
RandomJitter	Point cloud jitter, using Gaussian noise with sigma=0.005 and a clipping range of 0.02	Simulate noise interference during sensor acquisition and reduce the model’s overfitting risk to noisy data.
ElasticDistortion	Elastic distortion with parameters [[0.2, 0.4], [0.8, 1.6]]	Simulate natural morphological deformation during plant growth or slight deformation during acquisition and improve the model’s segmentation robustness for non-rigidly deformed targets.
SphereCrop	Spherical cropping, maximum number of points 80000, random mode	Control the number of input point clouds per batch, avoiding video memory overflow while retaining local feature details.

### 3.2 Ablation experiments

In the process of our experiments, the control variable method was adopted, and EL Loss, MRDCA, and PG-InvFR were integrated sequentially. By combining these three components, a total of eight ablation experiments were conducted to verify their effectiveness. The baseline model for these experiments is PTV2. The experimental results are presented in **Table 5**.

**Table 5:** Ablation experiment results.

Index	PTV2	+EL Loss	+MRDCA	+PG-InvFR	+EL Loss +MRDCA	+EL Loss +PG-InvFR	+MRDCA +PG-InvFR	PTV2-Fr
IoU	88.38	88.28	88.45	89.00	88.66	89.44	90.30	88.64
	79.28	79.40	79.44	79.81	80.08	80.66	82.33	83.48
	96.37	99.97	99.98	99.00	99.99	99.99	99.87	99.48
	88.01	89.22	89.29	89.27	89.58	90.03	90.83	90.53
Precision	96.23	97.98	97.59	96.77	96.29	97.21	98.84	94.66
	83.00	82.02	85.65	84.41	86.31	86.64	89.42	91.42
	96.37	99.99	99.98	99.99	99.99	99.99	99.93	99.66
	91.87	93.33	94.41	93.72	94.20	94.61	96.06	95.25
Recall	90.66	89.92	90.42	91.73	90.05	91.79	91.91	93.09
	94.61	96.13	95.34	93.60	96.72	94.50	93.88	90.71
	99.97	99.97	99.98	99.99	99.99	99.99	99.88	99.81
	95.08	95.34	95.25	95.11	95.59	95.43	95.22	94.54
F1-Score	93.83	93.78	93.87	94.18	93.99	94.42	95.30	93.86
	88.42	88.52	88.54	88.77	88.94	89.30	91.60	91.05
	98.15	99.99	99.99	99.99	99.99	99.99	99.93	99.73
	93.47	94.10	94.13	94.31	94.31	94.57	95.61	94.88

By comparing the experimental results of PTV2 and +MRDCA, mIoU increased by 1.28% and mP increased by 2.57%. This indicates that the MRDCA module can effectively capture multi-scale features and enhance coordinate attention, thereby improving the overall segmentation accuracy of the model. Comparing the baseline model with the +PG-InvFR, mIoU increased by 1.26%. This result shows that the PG-InvFR module can refine point features by generating adaptive weights based on neighborhood relationships, which is particularly beneficial for distinguishing fine-grained parts such as leaves and stems. By comparing the results of PTV2 and the +Loss, it is known that after integrating Lovász loss on the basis of cross-entropy loss, mIoU increased by 1.21%, which confirms that this composite loss strategy can directly optimize the segmentation metrics.

The results of all eight experimental groups confirm that the hybrid loss function, MRDCA module, and PG-InvFR module all make significant contributions to improving the model’s segmentation performance. Overall, the finally proposed PTV2-Fr model outperforms the baseline model PTV2 in most accuracy metrics: the mIoU increases by 2.52%, the mP rises by 3.38%, and the mF1 improves by 1.41%. Therefore, it can be concluded that in the organ segmentation task on this dataset, the PTV2-Fr model performs better than the baseline model PTV2.

To quantify not only the accuracy gain but also the computational footprint of each component, we report in **Table 6** the trainable parameters, peak GPU memory, and average inference time for all eight model variants in the ablation study. As shown in **Table 6**, MRDCA and PG-InvFR provide consistent improvements in segmentation performance with only modest increases in model size, memory usage, and latency, while the EL loss enhances boundary segmentation without affecting inference-time complexity. Overall, the final PTV2-Fr configuration achieves a favorable trade-off between segmentation accuracy and throughput for high-throughput phenotyping applications.

**Table 6:** Training hyperparameters used for baseline models and the proposed PTV2-Fr.

Metrics	PTV2	+EL LOSS	+MRDCA	+PG-InvFR	+EL LOSS +MRDCA	+EL LOSS + PG-InvFR	+MRDCA + PG-InvFR	PTV2-Fr
Params	3.96	3.96	3.94	3.96	3.94	3.96	3.94	3.94
Mem(MB)	1811.9	1811.9	1824.5	1812.0	1824.5	1812.0	1824.5	1824.5
Time	169.90	170.90	207.91	178.69	209.82	178.50	216.16	220.09

**Figure 4:**
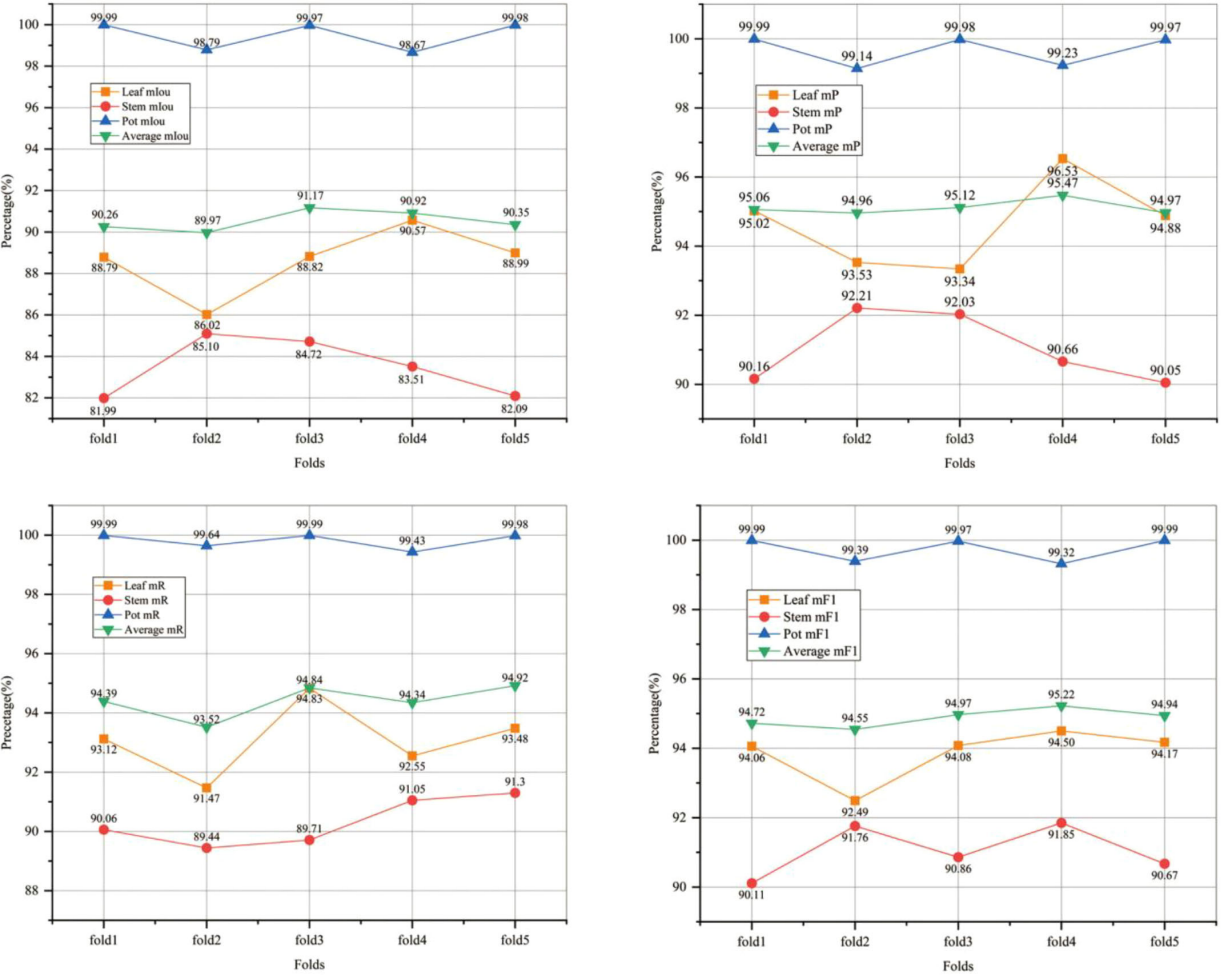
The results of k-fold cross-validation.

Furthermore, to evaluate model robustness, we employed a five-fold cross-validation protocol conducted at the pot level. Specifically, the set of pots was partitioned into five folds such that all point clouds and derived point blocks from the same pot were assigned to the same fold. In each iteration, four folds (≈80% of pots) were used for training and one fold (≈20% of pots) was used for validation; the final held-out test set remained completely independent of the cross-validation process. Cross-validation results are reported as mean ± standard deviation across the five folds for all main metrics to characterize result variability and model stability. The cross-validation outcomes are shown in **Figure 4**. To provide a statistical summary of model stability, the average performance is additionally reported as mean ± standard deviation across the five folds, as summarized in **Table 7**. The experimental results are shown in **Figure 4**.

**Table 7:** Mean ± standard deviation of five-fold cross-validation results for the PTV2-Fr model.

Metric	Mean(%)	Std(%)
mIou	90.53	0.46
mP	95.25	0.21
mR	94.54	0.56
mF1	94.88	0.26

The experimental results indicate that during the five-fold cross-validation process, PTV2-Fr exhibited stable performance across all metrics, with mIoU, mP, mR, and mF1 scores consistently maintained at high levels. The mIoU, mP, mR, and mF1 in the five experiments were 90.53%, 95.25%, 94.54%, and 94.88% respectively. Model generalization is evaluated using a strict pot-level hold-out test set, in which all test pots are completely unseen during training and cross-validation. Because each pot is scanned across multiple growth stages, the test set naturally includes unseen plant morphologies and developmental states, introducing a meaningful form of domain shift. This evaluation setup assesses the model’s ability to generalize to new physical plants and different growth stages, rather than claiming large-scale cross-domain generalization. These results demonstrate that PTV2-Fr performs excellently in the sorghum seedling point cloud organ segmentation task and exhibits strong generalization ability and stability under different data division methods. The application of the five-fold cross-validation method enables a more comprehensive evaluation of the model’s performance, reduces the risk of overfitting, and ensures the reliability and effectiveness of the model in practical applications.

**Figure 5:**
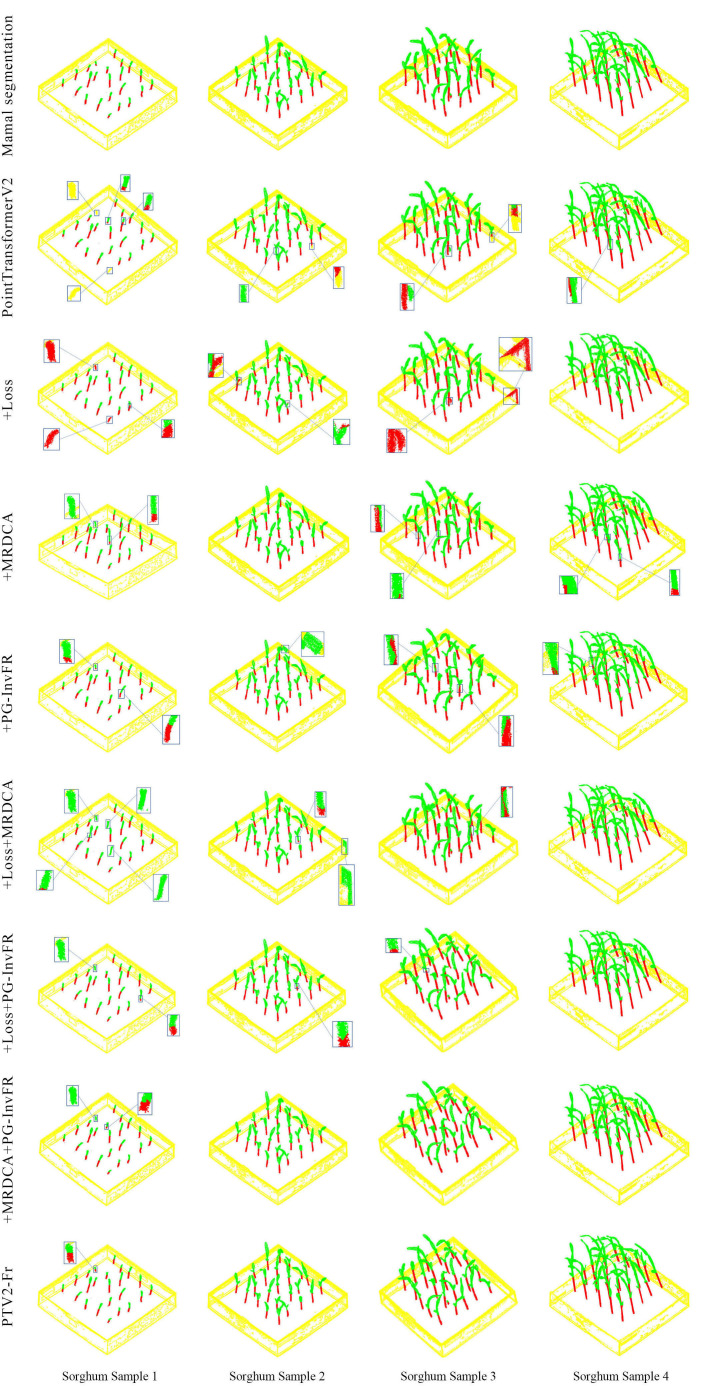
Qualitative visual analysis of sorghum seedlings in ablation experiments.

To visually evaluate the effectiveness of each submodule in the stem segmentation of sorghum seedling point clouds, four representative samples (A, B, C, and D) were selected from the test set for comparative visualization analysis, as shown in **Figure 5**. The figure presents the segmentation results of Manual segmentation, PTV2, the sequentially added modules, and the final model PTV2-Fr. In the visualization, red represents leaves, green represents stems, yellow indicates the pot, and the small boxes show enlarged details. As observed from the results, in samples A and B, the baseline model exhibits minor misclassification between leaves and stems and some omissions of fine stem segments. After introducing the Loss module, stem continuity was improved, the MRDCA module enhanced the recognition of curved structures, and the PG-InvFR module further improved the discrimination between stems and leaves. In sample C, where the leaves are more curved and overlapped, the +MRDCA module effectively alleviated segmentation gaps, while the +PG-InvFR module optimized global consistency, resulting in more complete segmentation. For the complex sample D, with dense and intertwined leaves and stems, PointTransformerV2 showed obvious misclassification and discontinuity. In contrast, the combination of multiple modules—especially +MRDCA+PG-InvFR and the final PTV2-Fr—accurately identified fine branches and maintained the integrity of stems and leaves, producing segmentation results close to the manual annotations. Overall, the Loss module improved local continuity, the MRDCA module strengthened multi-scale feature fusion to adapt to complex plant morphology, and the PG-InvFR module enhanced class discrimination. The integration of all three modules enabled the final model to outperform the baseline under varying densities and morphological conditions, achieving higher segmentation accuracy and completeness.

### 3.3 Comparison of point cloud semantic segmentation results for sorghum seedlings using different methods

Our study selected five common point cloud semantic segmentation networks—PointNet (Qi et al., 2017a), PointNet++ (Qi et al., 2017b), Point Transformer(PTV1) (Zhao et al., 2021), PTV3 (Wu et al., 2024), and U-Net (Çiçek et al., 2016)—to compare with PTV2-Fr. Section 3.1 has introduced the experimental settings and parameter configurations of PTV2-Fr, while the other networks adopted the recommended parameters in their original papers. For transparency and reproducibility, the exact training hyperparameters used for each baseline model and the proposed PTV2-Fr. are now explicitly reported in **Table 8**. **Table 9** presents the organ segmentation accuracy of the six networks, including IoU, Precision, Recall, and F1-Score.

**Table 8:** Training hyperparameters used for baseline models and the proposed PTV2-Fr.

Model	Optimizer	Learning rate	Weight decay	Batch size	Epochs	Scheduler
PointNet	Adam	0.001	/	16	200	Step
PointNet++	Adam	0.001	/	16	200	Step
PTV1	Adamw	0.005	0.01	16	400	Cosine
PTV3	Adamw	0.005	0.01	16	300	Cosine
U-Net	Adam	0.001	0.05	16	200	Step
PTV2-Fr	Adamw	0.001	0.05	32	100	MultiStepLR

**Table 9:** A comparative analysis of semantic segmentation performance across different deep learning networks.

Index	Part	PointNet	PointNet++	PTV1	PTV3	U-Net	PTV2-Fr
IoU	Leaf	84.21	87.91	86.80	83.63	89.03	88.64
	Stem	70.49	77.10	71.50	74.02	78.72	83.48
	Pot	82.71	98.68	88.48	89.91	90.69	99.48
	Mean	79.14	87.90	82.26	82.52	86.15	90.53
Precision	Leaf	94.00	95.46	95.93	98.61	95.29	94.66
	Stem	78.53	83.71	81.23	75.45	86.24	91.42
	Pot	83.31	99.02	88.60	89.92	90.69	99.66
	Mean	85.28	92.73	88.59	87.99	90.74	95.25
Recall	Leaf	88.96	91.74	90.12	84.63	93.13	93.09
	Stem	87.38	90.66	85.64	97.51	90.03	90.71
	Pot	99.17	99.69	99.80	99.92	99.99	99.81
	Mean	91.84	94.03	91.85	94.02	94.38	94.54
F1-Score	Leaf	91.41	93.56	92.94	91.09	94.20	93.86
	Stem	82.72	87.05	83.38	85.07	88.09	91.05
	Pot	90.55	99.35	93.90	94.69	95.12	99.73
	Mean	88.23	93.32	90.07	90.28	92.47	94.88

Early point cloud semantic segmentation models such as PointNet and PointNetV++ have laid the foundation for the processing of 3D point cloud data. Since PointNet only performs point-wise MLP and global pooling without neighborhood geometric modeling, it tends to confuse spatially close but semantically different points in the sorghum seedling semantic segmentation task. This results in relatively low comprehensive IoU values for leaf and stem classes. PointNet++ improves local modeling through hierarchical multi-scale aggregation, making it more capable of capturing local shapes and topology than pure global methods. Thus, it performs reliably when segmenting larger leaves or distinct stems. However, its performance is highly dependent on the settings of sampling radius and k-NN parameters. A single-scale configuration is prone to two types of errors on plant organs with variable scales and uneven point density: an excessively large radius leads to over-smoothing of boundaries, causing small leaves to be merged into stems; an overly small radius may fail to capture sufficient context, resulting in fragmentation or noise sensitivity. Overall, it exhibits over-smoothing or local underfitting when dealing with samples with large organ scale variations and uneven point density.

PTV1 leverages the self-attention mechanism of Transformer, introducing enhanced global feature extraction capability for point cloud processing. By modeling relationships between all points in the point cloud, it effectively aggregates long-range semantic information, thus demonstrating robustness in recognizing large-scale, distinct-shaped leaves or main stems and being able to correct local noise using global context. However, its original design does not focus on enhancing highly local geometric details—which leads to under-segmentation or errors of being merged into adjacent categories in structures with sparse points and complex topology, such as small leaves clinging to the main stem or newly emerged leaves. PTV3 introduces a unique design concept: it significantly expands the model’s effective receptive field and reduces real-time computation and memory overhead by replacing traditional KNN or ball queries with serialized neighborhood mapping. Nevertheless, this does not solve the problem of being merged into adjacent categories when target objects are extremely small, have sparse point counts, or are highly geometrically similar to neighboring structures. Without additional local refinement or multi-scale compensation, serialized neighborhood aggregation tends to smooth out key local discriminative features in attention allocation.

U-Net, which is based on spconv, uses voxelization as a preprocessing step, converting the point cloud into a sparse voxel grid. When the voxel size is relatively coarse, small targets with fewer points and limited spatial extent—such as petioles, small leaves attached to the main stem, or the pot—may be merged into neighboring regions, resulting in information loss or blurred boundaries. This is the primary reason for the low IoU of small classes. Moreover, the downsampling and upsampling operations in the sparse U-Net, together with submanifold convolutions, tend to smooth details during multiple aggregation processes. Without additional point-level local refinement, the smoothed information becomes difficult to recover, causing small objects to be merged into adjacent classes or to appear broken.

Experimental results show that PTV2-Fr outperforms recent point cloud segmentation models in overall performance, achieving 90.53% mIoU, 95.25% mPrec, 94.54% mRec, and 94.88% mF1 in the sorghum seedling point cloud organ segmentation task. Among the advanced models listed, PTV2-Fr is the most suitable model for segmenting sorghum seedling point clouds.

**Figure 6:**
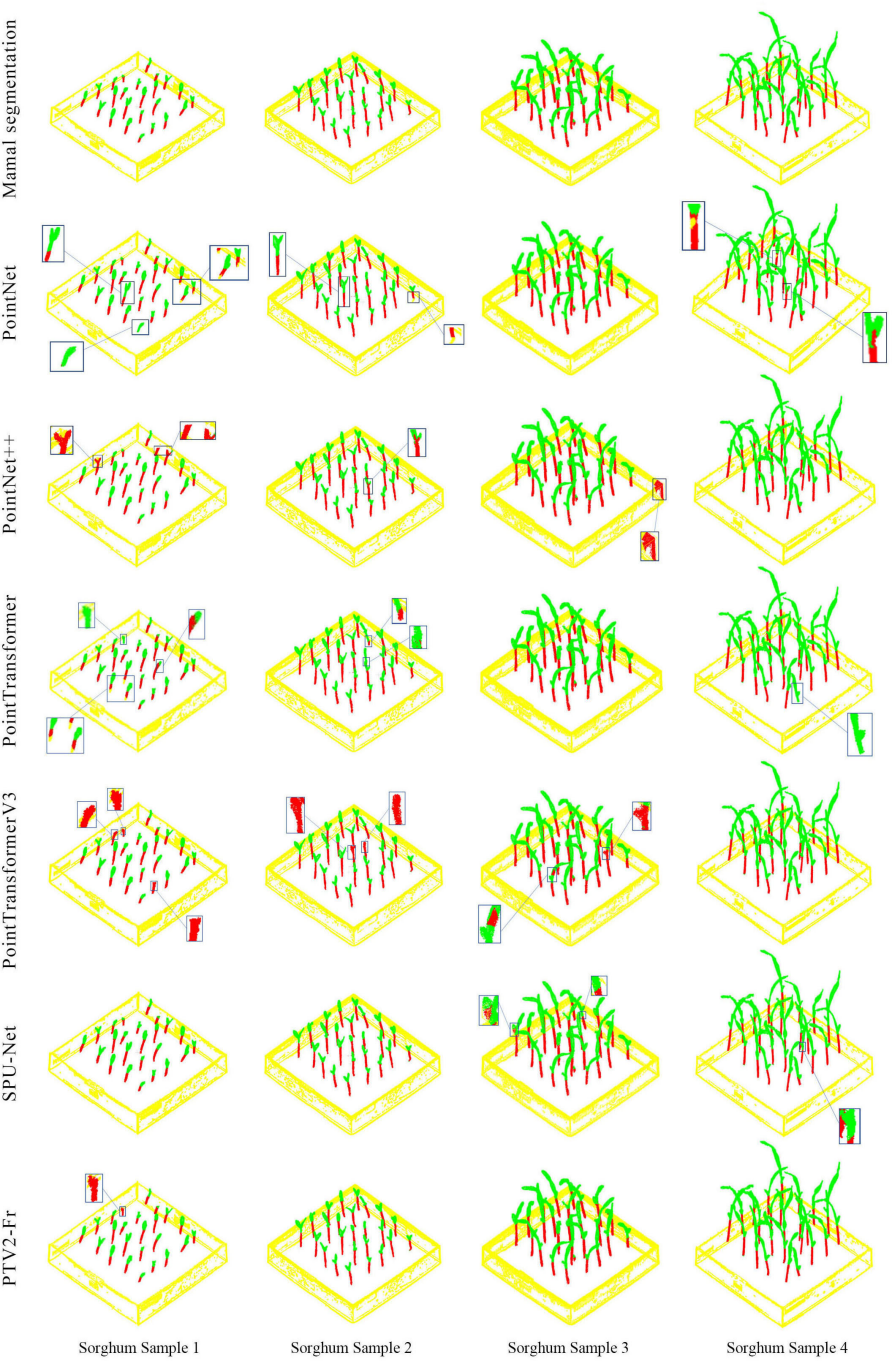
Qualitative visual analysis of sorghum seedlings in different experiments.

**Figure 6** presents a visual comparison of different methods for stem segmentation in sorghum seedling point clouds, using four representative samples (A, B, C, and D) for analysis. The figure includes the results of Manual segmentation, PointNet, PointNet++, PTV1, PTV3, U-Net, and the proposed PTV2-Fr model. In the visualization, red represents leaves, green represents stems, yellow represents the pot, and the small boxes show enlarged details of local segmentation areas.

Overall, the traditional methods PointNet and PointNet++ exhibit relatively low segmentation accuracy, often showing confusion between leaves and stems as well as stem discontinuities. Due to its simple feature extraction mechanism, PointNet displays obvious misclassifications in samples A and B, with fine stem segments being incorrectly identified as leaves. Although PointNet++ enhances local feature representation through a hierarchical structure, it still suffers from segmentation breaks and omissions in the more complex samples C and D, particularly in regions where stems and leaves are intertwined. The introduction of the attention mechanism in PTV1 improves local segmentation consistency to some extent, making the boundary between stems and leaves clearer. However, in dense samples (such as C and D), some misclassifications and stem breaks remain observable. PTV3 further refines feature modeling, achieving better overall performance and distinguishing stems and leaves more effectively, though minor errors still occur in curved and overlapping structures.

U-Net performs well in multi-scale feature fusion, achieving stable segmentation of stems and leaves under complex morphological conditions. The enlarged details show that U-Net significantly improves stem continuity and leaf boundary delineation. In comparison, the proposed PTV2-Fr integrates multi-module feature fusion and structure-adaptive mechanisms, yielding segmentation results most consistent with manual annotations. Whether in sparse (A, B) or dense and complex (C, D) samples, PTV2-Fr accurately identifies fine stem segments while maintaining structural integrity, producing clear leaf–stem boundaries and significantly reducing misclassification.

In summary, the visual results demonstrate that segmentation accuracy and stability improve progressively with model refinement. By integrating multi-level feature representations with global contextual information, PTV2-Fr achieves the best performance among all methods in the challenging sorghum seedling point cloud segmentation task. It effectively addresses the segmentation difficulties caused by leaf curvature, overlap, and complex morphology, resulting in more accurate and complete stem–leaf segmentation outcomes.

### 3.4 Detection of sorghum seedling phenotypes under different concentrations of GA_3_ solution

**Figure 7:**
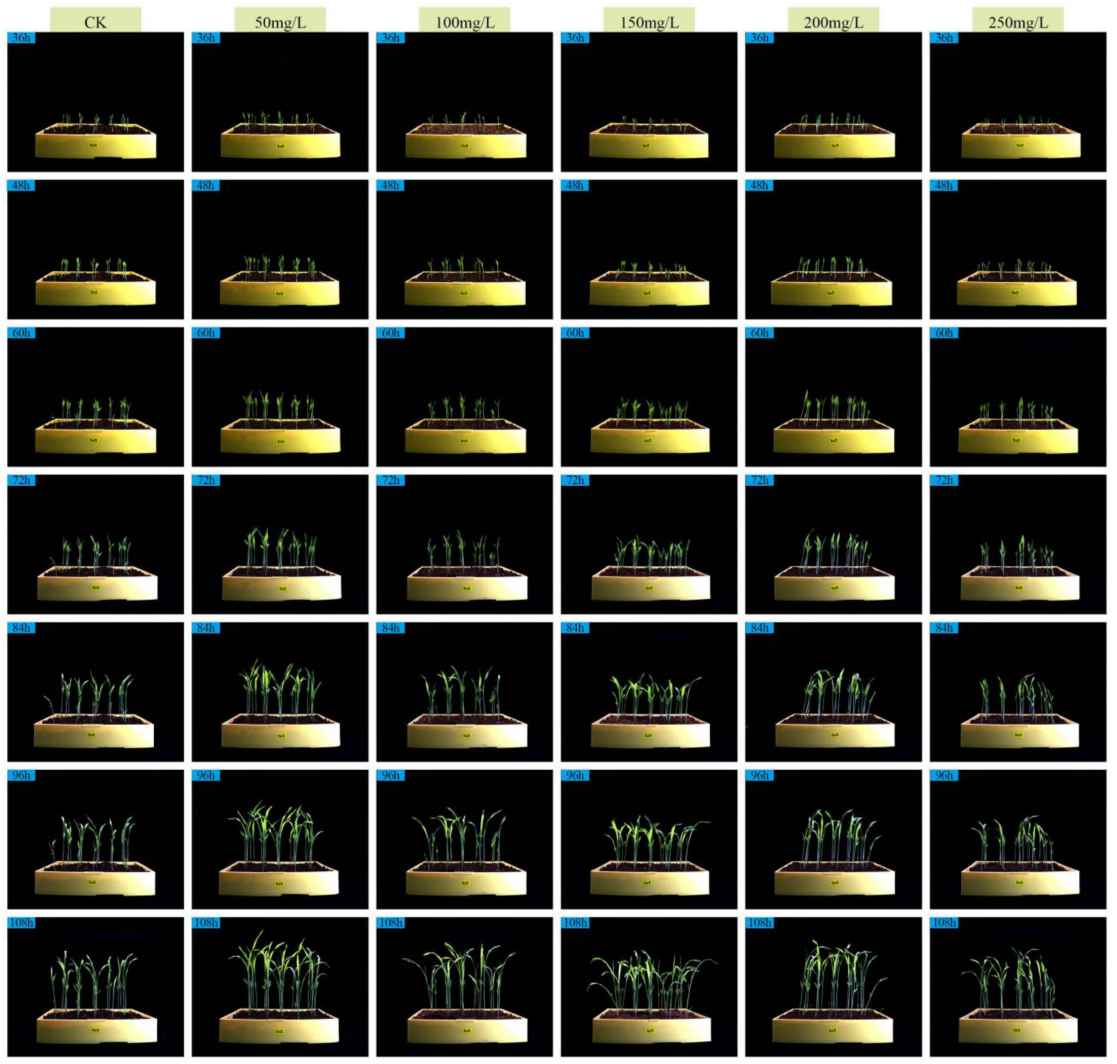
Front-view captured images of sorghum seedlings during the growth process.

**Figure 8:**
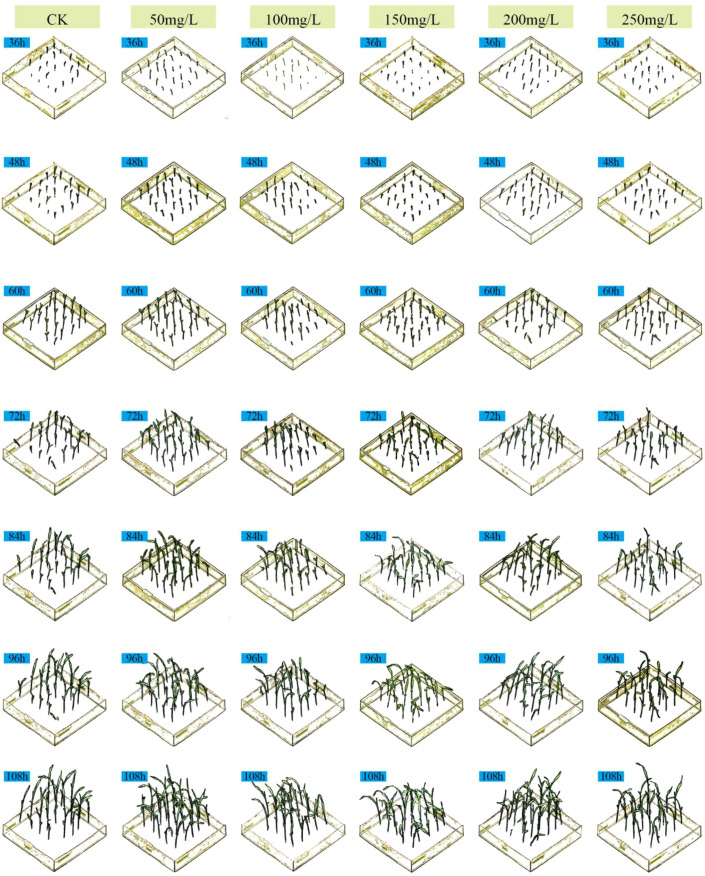
Three-view point cloud reconstructed images of sorghum seedlings during the growth process.

**Figure 9:**
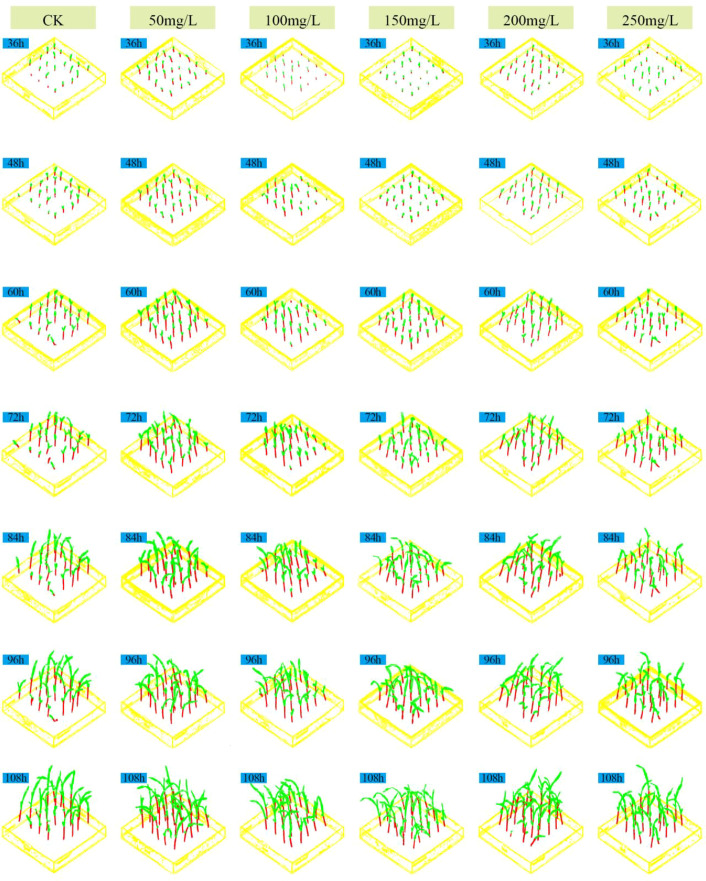
Semantic segmentation of point cloud data images of sorghum seedlings using the PTV2-Fr model.

**Figure 10:**
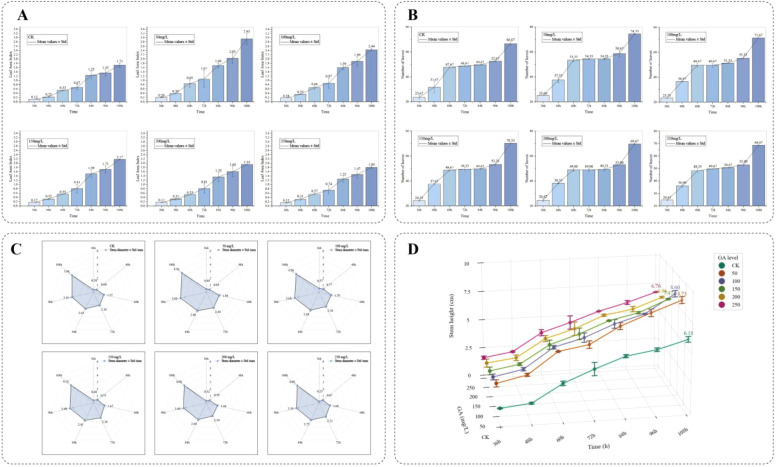
**(A)** The changes in the total number of leaves of sorghum seedlings over time in the CK and different concentrations of GA_3_ solutions; **(B)** The changes in the LAI of sorghum seedlings over time in the CK and different concentrations of GA_3_ solutions; **(C)** The changes in the average axial stem height of sorghum seedlings over time in the CK and different concentrations of GA_3_ solutions; **(D)** The changes in the average stem diameter of sorghum seedlings over time in the CK and different concentrations of GA_3_ solutions.

Numerous existing studies have indicated that GA_3_ within an appropriate range can promote germination and seedling elongation, but a dose-response phenomenon of “low-concentration promotion and high-concentration inhibition” may occur at excessively high concentrations or under specific stress scenarios. Based on the hypothesis of this hormone dose-response, our study took GA_3_ as the treatment factor to systematically examine the threshold effect of different concentrations on the growth performance of sorghum seedlings at the seedling stage. For this purpose, six treatment groups were set up: control check (CK) consisting of deionized water, and 50, 100, 150, 200, and 250 mg·L^-^¹ GA_3_ solutions. Three-dimensional (3D) point cloud data were collected every 12 hours within the range of 36 h to 108 h after sowing. Because the same physical pot was scanned repeatedly over time (i.e., longitudinal sampling), care was taken during dataset partitioning to avoid temporal leakage: all scans from a single pot were assigned to the same data subset (training, validation, or test). This ensures that temporal measurements from the same plant never appear across different evaluation subsets. **Figure 7** shows the front-view images of sorghum seedlings during growth, **Figure 8** presents the 3D point cloud reconstruction images of sorghum seedlings during growth, and **Figure 9** displays the semantic segmentation results of sorghum seedling 3D point cloud data using the PTV2-Fr model. For the 3D point cloud data after semantic segmentation, a clustering segmentation method was used to automatically identify and quantify the number of leaves. Axial stem height, leaf area, and basal stem diameter were extracted from the segmented voxels. Three replicate experiments were conducted for each treatment to calculate the average growth rate and relative response value. **Figure 10** shows the variations in the total number of leaves, leaf area index(LAI), average axial stem height, and average stem diameter of sorghum seedlings under the CK and different concentrations of GA_3_ solutions. To analyze the effects of GA_3_ concentration on sorghum seedling phenotypic traits while accounting for repeated measurements over time, a linear mixed-effects model (LMM) was employed. In the model, GA_3_ concentration, measurement time, and their interaction were treated as fixed effects, while pot identity was included as a random effect to account for within-pot correlations arising from repeated measurements. Statistical significance was evaluated at the 0.05 level.

(**Figure 10A**) shows the changes in the total number of leaves of sorghum seedlings over time in the CK and different concentrations of GA_3_ solutions. With the passage of time, the total number of leaves increased in both CK and all GA_3_-treated groups. However, as the concentration gradient of solution increased, the total number of leaves first increased and then decreased, reaching a peak at 50 mg/L. At the 7th recording (i.e., 108 h after sowing), the total number of leaves in CK, 50 mg/L, 100 mg/L, 150 mg/L, 200 mg/L, and 250 mg/L groups were 66.67, 74.33, 71.67, 70.33, 69.67, and 68.67, respectively. Overall, within the concentration range of 50 mg/L to 250 mg/L, the promoting effect of GA_3_ on the total number of leaves of sorghum seedlings first strengthened and then weakened, with the strongest effect observed at 50 mg/L. LMM revealed a significant main effect of GA_3_ concentration (p< 0.001) and time (p< 0.001), as well as a significant GA_3_ concentration × time interaction (p< 0.01). Estimated marginal means indicated that the 50 mg/L treatment produced a higher leaf number than the control (mean difference = 7.66, 95% CI [5.21, 10.11]).

(**Figure 10B**) shows the changes in the LAI of sorghum seedlings over time in the CK and different concentrations of GA_3_ solutions. Since the average leaf area may be inaccurate due to the influence of newly emerged leaves, we adopted LAI as the indicator, with the formula: total leaf area divided by unit area (625 cm²). With the passage of time, the LAI increased in both CK and all GA_3_-treated groups. However, as the concentration gradient of GA_3_ solution increased, the LAI first increased and then decreased, reaching a peak at 50 mg/L. At the 7th recording, the LAI values in CK, 50 mg/L, 100 mg/L, 150 mg/L, 200 mg/L, and 250 mg/L groups were 1.71, 2.93, 2.44, 2.17, and 1.80, respectively. Overall, within the concentration range of 50 mg/L to 250 mg/L, the promoting effect of GA_3_ on the LAI of sorghum seedlings first strengthened and then weakened, with the strongest effect observed at 50 mg/L. LMM revealed a significant main effect of GA_3_ concentration (p< 0.001) and time (p< 0.001), as well as a significant GA_3_ concentration × time interaction (p< 0.01). Estimated marginal means indicated that the 50 mg/L treatment produced a higher LAI than the control (mean difference = 1.22, 95% CI [0.89, 1.55]).

(**Figure 10C**) shows the changes in the average axial stem height of sorghum seedlings over time in the CK and different concentrations of GA_3_ solutions. With the passage of time, the average axial stem height increased in both CK and all GA_3_-treated groups. However, as the concentration gradient of GA_3_ solution increased, the average axial stem height first increased and then decreased, reaching a peak at 50 mg/L. At the 7th recording the average axial stem height values in CK, 50 mg/L, 100 mg/L, 150 mg/L, 200 mg/L, and 250 mg/L groups were 3.98cm, 4.53cm, 4.28cm, 4.15cm, 4.09cm, and 4.08cm, respectively. Overall, within the concentration range of 50 mg/L to 250 mg/L, the promoting effect of GA_3_ on the average axial stem height of sorghum seedlings first strengthened and then weakened, with the strongest effect observed at 50 mg/L. LMM revealed a significant main effect of GA_3_ concentration (p< 0.05) and time (p< 0.001), as well as a significant GA_3_ concentration × time interaction (p< 0.05). Estimated marginal means indicated that the 50 mg/L treatment produced a larger stem diameter than the control (mean difference = 0.55, 95% CI [0.32, 0.78]).

(**Figure 10D**) shows the changes in the average stem diameter of sorghum seedlings over time in the CK and different concentrations of GA_3_ solutions. With the passage of time, the average stem diameter increased in both CK and all GA_3_-treated groups. However, as the concentration gradient of GA_3_ solution increased, the average stem diameter first increased and then decreased, reaching a peak at 50 mg/L. At the 7th recording, the average stem diameter values in CK, 50 mg/L, 100 mg/L, 150 mg/L, 200 mg/L, and 250 mg/L groups were 6.18mm, 8.73mm, 8.60mm, 7.47mm, 6.96mm, and 6.76mm, respectively. Overall, within the concentration range of 50 mg/L to 250 mg/L, the promoting effect of GA_3_ on the average stem diameter of sorghum seedlings first strengthened and then weakened, with the strongest effect observed at 50 mg/L. LMM revealed a significant main effect of GA_3_ concentration (p< 0.001) and time (p< 0.001), as well as a significant GA_3_ concentration × time interaction (p< 0.001). Estimated marginal means indicated that the 50 mg/L treatment produced a taller stem height than the control (mean difference = 2.55, 95% CI [1.98, 3.12]).

GA_3_ affects seedling growth through multiple interacting physiological pathways.

Firstly, GA_3_ promotes cell elongation—primarily by inducing cell wall relaxation-related enzymes/proteins (e.g., expansin, XET) to reduce cell wall rigidity, thereby promoting longitudinal internode elongation rather than increasing the number of internodes. This type of cell wall relaxation mechanism is an important basis for elongation-promoting responses in plants (Cosgrove, 2024).

Secondly, GA_3_ can induce the mobilization of storage substances in seeds/endosperms (e.g., inducing α-amylase synthesis), accelerating the decomposition of starch into soluble sugars to provide carbon sources and energy for rapid growth (Hedden, 2025).

Thirdly, in terms of antioxidant and stress response, appropriate concentrations of GA_3_ can increase the activities of enzymes such as SOD, POD, CAT, and APX, thereby reducing the accumulation of ROS and MDA, and maintaining membrane system and cellular homeostasis. This is of great significance for seedling protection under stress conditions (Shahzad et al., 2021).

Fourthly, appropriate concentrations of GA_3_ can increase chlorophyll content, net photosynthetic rate, and stomatal conductance, while decreasing intercellular CO_2_ concentration. These changes indicate higher carbon assimilation capacity and gas exchange efficiency, providing energy and material basis for biomass accumulation. In contrast, excessive hormones or hormonal imbalance may be accompanied by photosynthetic inhibition and chlorophyll degradation, thereby impairing growth advantages (Fu et al., 2023).

In addition, under stress conditions such as salt stress, GA_3_ has been reported to be involved in regulating ion homeostasis (e.g., reducing intercellular Na^+^, increasing K^+^/Ca²^+^ ratios), synergizing with the aforementioned pathways to alleviate salt damage (Ali et al., 2021); however, the dose and application method determine its positive and negative effects. Combined with the “first increase and then decrease” response observed in this study’s gradient experiment of 50–250 mg·L^-^¹ (with 50 mg·L^-^¹ as the optimal concentration), it can be concluded that low to medium concentrations of GA_3_ mainly promote seedling growth by enhancing cell elongation, nutrient mobilization, antioxidant capacity, and photosynthetic capacity, while high concentrations may cause inhibition due to hormonal signal and metabolic disorders.

In summary, within the concentration range of 50 mg/L to 250 mg/L, the promoting effect of GA_3_ on the growth of sorghum seedlings first strengthened and then weakened, with the maximum effect observed at 50 mg/L.

## 4 Conclusions

Our study proposes and implements a point cloud semantic segmentation network PTV2-Fr for organ segmentation and automatic extraction of phenotypic parameters of sorghum seedlings at the seedling stage. These early-stage phenotypic traits, such as leaf number, leaf area index, and stem height, are widely used as indicators of seedling vigor and establishment capacity, which are important targets in early-stage selection and screening in sorghum breeding programs. In terms of structural design, PTV2-Fr makes three key improvements targeting the characteristics of seedling point clouds: MRDCA for multi-scale geometric and coordinate perception enhancement; PG-InvFR for refining boundaries and local features before the segmentation head; and the composite loss EL Loss for alleviating class imbalance and directly optimizing IoU. Based on the sorghum seedling point cloud dataset constructed and manually annotated by us, systematic experiments and ablation studies show that the above designs make significant contributions to improving organ segmentation accuracy and boundary robustness.

PTV2-Fr demonstrates robust performance in quantitative evaluation: compared with the baseline PointTransformerV2, the overall accuracy is improved by approximately 2.5%; in terms of key segmentation metrics, the model achieves a mIoU increase of about 2.52%, a mPrec improvement of around 3.38%, and a mF1 rise of roughly 1.41% compared to the baseline. It should be noted that the objective of this study is not to directly predict final yield or late-stage agronomic performance, but to provide a reliable and fine-grained phenotyping tool for early-stage screening and comparative analysis under controlled experimental conditions. Ablation experiments further confirm the positive contributions of the three modules (MRDCA, PG-InvFR, and EL Loss) to performance—each brings significant improvements in mIoU and boundary accuracy under different combinations. When compared with other common point cloud segmentation networks (PointNet, PointNet++, PTV1, PTV3, and U-Net), PTV2-Fr exhibits superior comprehensive performance in scenarios with dense seedlings, large differences in organ scales, and severe local occlusion, enabling more accurate distinction between three semantic regions: stems, leaves, and flowerpots.

While our model achieves high performance on the sorghum seedling dataset, there are still several limitations that need to be addressed. One significant challenge is handling occlusion. In plant point cloud data, occlusion often occurs when leaves or stems overlap, causing parts of the plant to be obscured. This issue can lead to missing or inaccurate segmentation of plant organs. To address this, future work could explore the integration of multi-view data, depth sensing, or point cloud completion techniques, which can help fill in the gaps caused by occlusion.

Another limitation is lighting changes, which can significantly impact point cloud quality. Variations in lighting conditions, such as changes in ambient light or sensor positioning, may affect the reflectivity and quality of point cloud data. While deep learning models show robustness under controlled conditions, their performance may degrade in real-world scenarios with varying lighting. Future research could focus on augmenting training data with varied lighting conditions or applying domain adaptation techniques to make models more resilient to lighting changes.

Finally, cross-species generalization remains a challenge. Our model was trained and evaluated on sorghum seedlings, and its performance on other plant species is not fully known. Different plant species have varying organ shapes, sizes, and structures, which can lead to difficulties in generalizing across species. To improve generalization, future work could focus on developing more robust models that are capable of handling a variety of plant species, potentially through the use of multi-species datasets and transfer learning techniques.

Based on the above limitations, future research can carry out improvements and expansions in the following directions: First, introduce multi-modal data and design a cross-modal feature alignment module to enhance stability under occlusion and light changes. Second, construct a multi-crop joint training or transfer learning framework to adapt the model to crops with significant morphological differences such as corn and wheat, and improve cross-species generalization ability. Third, it is necessary to extend the approach to large-scale, high-throughput data collection in field environments, including outdoor and semi-field conditions, in order to evaluate the model’s applicability in real agricultural production scenarios. Fourth, combine uncertainty estimation with active acquisition strategies to trigger supplementary collection or manual review on samples with high uncertainty, thereby improving the system’s reliability and deploy ability.

In conclusion, PTV2-Fr provides a feasible and high-performance technical solution for the efficient, non-destructive, and automated measurement of sorghum seedling phenotypes at the seedling stage. It not only significantly improves the recognition ability for small targets and boundaries in point cloud organ segmentation tasks but also offers reliable tool support for large-scale agricultural phenotypic quantification and studies on hormonal treatment responses based on point clouds. Once trained, the proposed model enables automated, non-destructive phenotypic measurement with minimal human intervention, which makes the pipeline suitable for scaling to larger populations in breeding and physiological studies. In the future, through data scale expansion, multi-modal fusion, and cross-crop generalization research, PTV2-Fr is expected to promote the application implementation of point cloud-driven high-throughput crop phenomics in breeding and precision cultivation.

## Data Availability

The raw data supporting the conclusions of this article will be made available by the authors, without undue reservation.

## References

[B1] Ali A. Y. A. Ibrahim M. E. H. Zhou G. Nimir N. E. A. Elsiddig A. M. I. Jiao X. . (2021). Gibberellic acid and nitrogen efficiently protect early seedlings growth stage from salt stress damage in sorghum. Sci. Rep. 11, 6672. doi: 10.1038/s41598-021-84713-9, PMID: 33758238 PMC7988071

[B2] Berman M. Triki A. R. Blaschko M. B. (2018). “ The Lovász-Softmax loss: A tractable surrogate for the optimization of the intersection-over-union measure in neural networks,” in Proc. IEEE Conf. Comput. Vis. Pattern Recognit (Salt Lake City, UT, USA: IEEE), pp. 4413–4421. doi: 10.1109/CVPR.2018.00464

[B3] Boogaard F. P. van Henten E. J. Kootstra G. (2022). Improved point-cloud segmentation for plant phenotyping through class-dependent sampling of training data to battle class imbalance. Front. Plant Sci. 13. doi: 10.3389/fpls.2022.838190, PMID: 35419014 PMC8996061

[B4] Chu M. De Maria G. L. Dai R. Benenati S. Yu W. Zhong J. . (2024). DCCAT: Dual-coordinate cross-attention transformer for thrombus segmentation on coronary OCT. Med. Image Anal. 97, 103265. doi: 10.1016/j.media.2024.103265, PMID: 39029158

[B5] Çiçek Ö. Abdulkadir A. Lienkamp S. S. Brox T. Ronneberger O. (2016). 3D U-Net: Learning dense volumetric segmentation from sparse annotation. Medical Image Computing and Computer-Assisted Intervention – MICCAI 2016, Lecture Notes in Computer Science 9901, 424–432. doi: 10.1007/978-3-319-46723-8_49

[B6] Cosgrove D. J. (2024). Plant cell wall loosening by expansins. Annu. Rev. Cell Dev. Biol. 40, 329–352. doi: 10.1146/annurev-cellbio-111822-115334, PMID: 38724021

[B7] Deng Q. Zhao J. Li R. Liu G. Hu Y. Ye Z. . (2024). A precise segmentation algorithm of pumpkin seedling point cloud stem based on CPHNet. Plants 13, 2300. doi: 10.3390/plants13162300, PMID: 39204736 PMC11359360

[B8] Du R. Zhai G. Qiu T. Jiang Y. (2025). Towards scalable organ level 3D plant segmentation: bridging the data algorithm computing gap. (Ithaca, NY, USA: Cornell University). doi: 10.48550/arXiv.2509.06329

[B9] Fu J. Li L. Wang S. Yu N. Shan H. Shi Z. . (2023). Effect of gibberellic acid on photosynthesis and oxidative stress response in maize under weak light conditions. Front. Plant Sci. 14. doi: 10.3389/fpls.2023.1128780, PMID: 36875610 PMC9978513

[B10] Galba A. Masner J. Kholová J. Kartal S. Stočes M. Mikeš V. . (2025). Annotated 3D point cloud dataset of broad−leaf legumes captured by high−throughput phenotyping platform. Sci. Data 12, 1764. doi: 10.1038/s41597-025-06049-7, PMID: 41213977 PMC12603112

[B11] Gao T. Zhu F. Paul P. Sandhu J. Doku H. A. Sun J. . (2021). Novel 3D imaging systems for high-throughput phenotyping of plants. Remote Sens. 13, 2113. doi: 10.3390/rs13112113

[B12] Gilson A. Meyer L. Scholz O. Schmid U. (2025). OmniPlantSeg: Species−agnostic 3D point cloud organ segmentation for high−resolution plant phenotyping across modalities. arXiv [preprint]. arXiv:2509.21038. doi: 10.48550/arXiv.2509.21038

[B13] Golbach F. Kootstra G. Damjanovic S. Otten G. van de Zedde R. (2016). Validation of plant part measurements using a 3D reconstruction method suitable for high-throughput seedling phenotyping. Mach. Vis. Appl. 27, 663–680. doi: 10.1007/s00138-015-0727-5

[B14] Gupta S. Tripathi A. K. (2025). Flora-NET: Integrating dual coordinate attention with adaptive kernel based convolution network for medicinal flower identification. Comput. Electron. Agric. 230, 109834. doi: 10.1016/j.compag.2024.109834

[B15] Harandi N. Vandenberghe B. Vankerschaver J. Depuydt S. Van Messem A. (2023). How to make sense of 3D representations for plant phenotyping: A compendium of processing and analysis techniques. Plant Methods 19, 60. doi: 10.1186/s13007-023-01031-z, PMID: 37353846 PMC10288709

[B16] Hedden P. (2025). Induction of α-amylase and endosperm-imposed seed dormancy: Two pioneering papers in gibberellin research. Planta 261, 118. doi: 10.1007/s00425-025-04699-w, PMID: 40278915 PMC12031936

[B17] Heiwolt K. Duckett T. Cielniak G. (2021). Deep semantic segmentation of 3D plant point clouds. Toward Auton. Robot. Syst. 13054, 36–45. doi: 10.1007/978-3-030-89177-0_4

[B18] Hossain M. S. Islam M. N. Rahman M. M. Mostofa M. G. Khan M. A. R. (2022). Sorghum: A prospective crop for climatic vulnerability, food and nutritional security. J. Agric. Food Res. 8, 100300. doi: 10.1016/j.jafr.2022.100300

[B19] Jin S. Li D. Yun T. Tang J. Wang K. Li S. . (2025). Deep learning for three−dimensional (3D) plant phenomics. Plant Phenomics 7, 100107. doi: 10.1016/j.plaphe.2025.100107 PMC13109328 42040998

[B20] Jingwen W. Hong L. (2012). Measurement and analysis of plant leaf area based on image processing. Proc. Int. Symp. Inf. Technol. Med. Educ. 2, 1070–1074. doi: 10.1109/ITIME.2012.6291485

[B21] Koyama K. (2023). Leaf area estimation by photographing leaves sandwiched between transparent clear file folder sheets. Horticulturae 9, 709. doi: 10.3390/horticulturae9060709

[B22] Li D. Shi G. Li J. Chen Y. Zhang S. Xiang S. . (2022). PlantNet: A dual-function point cloud segmentation network for multiple plant species. ISPRS J. Photogramm. Remote Sens. 184, 243–263. doi: 10.1016/j.isprsjprs.2022.01.007

[B23] Liu Z. Zhao J. Hu Y. Li R. Deng Q. Guan R. . (2025). FACNet: A high-precision pumpkin seedling point cloud organ segmentation method. Comput. Electron. Agric. 231, 110049. doi: 10.1016/j.compag.2025.110049

[B24] Mertoğlu K. Şalk Y. Sarıkaya S. K. Turgut K. Evrenesoğlu Y. Çevikalp H. . (2024). PLANesT-3D: A new annotated dataset for segmentation of 3D plant point clouds. arXiv [preprint] arXiv:2407.21150 doi: 10.48550/arXiv.2407.21150

[B25] Miao T. Zhu C. Xu T. Yang T. Li N. Zhou Y. . (2021). Automatic stem-leaf segmentation of maize shoots using three-dimensional point cloud. Comput. Electron. Agric. 187, 106310. doi: 10.1016/j.compag.2021.106310

[B26] Mwamahonje A. Mdindikasi Z. Mchau D. Mwenda E. Sanga D. Garcia-Oliveira A. L. . (2024). Advances in sorghum improvement for climate resilience in the global arid and semi-arid tropics: A review. Agronomy 14, 3025. doi: 10.3390/agronomy14123025

[B27] Nguyen T. T. Slaughter D. C. Max N. Maloof J. N. Sinha N. (2015). Structured light-based 3D reconstruction system for plants. Sensors 15, 18587–18612. doi: 10.3390/s150818587, PMID: 26230701 PMC4570338

[B28] Paterson A. H. Bowers J. E. Bruggmann R. Dubchak I. Grimwood J. Gundlach H. . (2009). The *Sorghum bicolor* genome and the diversification of grasses. Nature 457, 551–556. doi: 10.1038/nature07723, PMID: 19189423

[B29] Qi C. R. Su H. Mo K. Guibas L. J. (2017a). “ PointNet: Deep learning on point sets for 3D classification and segmentation,” in Proc. IEEE Conf. Comput. Vis. Pattern Recognit Honolulu, HI, USA (Piscataway, NJ: IEEE). 77–85. doi: 10.1109/CVPR.2017.16

[B30] Qi C. R. Yi L. Su H. Guibas L. J. (2017b). PointNet++: Deep hierarchical feature learning on point sets in a metric space. Adv. Neural Inf. Process. Syst. (Red Hook, NY: Curran Associates, Inc.) 30, 4–9. doi: 10.48550/arXiv.1706.02413

[B31] Shahzad K. Hussain S. Arfan M. Hussain S. Waraich E. A. Zamir S. . (2021). Exogenously applied gibberellic acid enhances growth and salinity stress tolerance of maize through modulating the morpho-physiological, biochemical and molecular attributes. Biomolecules 11, 1005. doi: 10.3390/biom11071005, PMID: 34356629 PMC8301807

[B32] Shen J. Wu T. Zhao J. Wu Z. Huang Y. Gao P. . (2024). Organ segmentation and phenotypic trait extraction of cotton seedling point clouds based on a 3D lightweight network. Agronomy 14, 1083. doi: 10.3390/agronomy14051083

[B33] Song H. Wen W. Wu S. Guo X. (2025). Comprehensive review on 3D point cloud segmentation in plants. Artif. Intell. Agric. 15, 296–315. doi: 10.1016/j.aiia.2025.01.006

[B34] Tu L.-F. Peng Q. Li C.-S. Zhang A. (2021). 2D in *situ* method for measuring plant leaf area with camera correction and background color calibration. Sci. Program. 2021, 6650099. doi: 10.1155/2021/6650099

[B35] Vayssade J.-A. Jones G. Gée C. Paoli J.-N. (2022). Pixelwise instance segmentation of leaves in dense foliage. Comput. Electron. Agric. 195, 106797. doi: 10.1016/j.compag.2022.106797

[B36] Wang R.-F. Qu H.-R. Su W.-H. (2025). From sensors to insights: Technological trends in image-based high-throughput plant phenotyping. Smart Agric. Technol. 12, 101257. doi: 10.1016/j.atech.2025.101257

[B37] Wu X. Jiang L. Wang P.-S. Liu Z. Liu X. Qiao Y. . (2024). “ Point transformer v3: Simpler, faster, stronger,” in Proc. IEEE/CVF Conf. Comput. Vis. Pattern Recognit (Piscataway, NJ: IEEE). doi: 10.1109/CVPR52733.2024.00463

[B38] Xiang L. Tang L. Gai J. Wang L. (2021). Measuring stem diameter of sorghum plants in the field using a high-throughput stereo vision system. Trans. ASABE 64, 1999–2010. doi: 10.13031/trans.14156

[B39] Zhang Y. Xie Y. Zhou J. Xu X. Miao M. (2024). Cucumber seedling segmentation network based on a multiview geometric graph encoder from 3D point clouds. Plant Phenomics 6, 254. doi: 10.34133/plantphenomics.0254, PMID: 39415968 PMC11480588

[B40] Zhao H. Jiang L. Jia J. Torr P. H. S. Koltun V. (2021). “ Point transformer,” in Proc. IEEE/CVF Int. Conf. Comput. Vis (Piscataway, NJ: IEEE). doi: 10.1109/ICCV48922.2021.01595

[B41] Zhou J. Applegate C. Alonso A. D. Reynolds D. Orford S. Mackiewicz M. . (2017). Leaf-GP: An open and automated software application for measuring growth phenotypes for *Arabidopsis* and wheat. Plant Methods 13, 117. doi: 10.1186/s13007-017-0266-3, PMID: 29299051 PMC5740932

[B42] Zhou Y. Qi Y. Xiang L. (2025). Automatic extraction method of phenotypic parameters for *Phoebe zhennan* seedlings based on 3D point cloud. Agriculture 15, 834. doi: 10.3390/agriculture15080834

[B43] Zhou W. Wang X. Yang X. Hu Y. Yi Y. (2024). Skeleton-guided multi-scale dual-coordinate attention aggregation network for retinal blood vessel segmentation. Comput. Biol. Med. 181, 109027. doi: 10.1016/j.compbiomed.2024.109027, PMID: 39178808

[B44] Zhu J. Zhai R. Ren H. Xie K. Du A. He X. . (2024). Crops3D: A diverse 3D crop dataset for realistic perception and segmentation toward agricultural applications. Sci. Data 11, 1438. doi: 10.1038/s41597-024-04290-0, PMID: 39730336 PMC11681092

